# The kynurenine pathway presents multi-faceted metabolic vulnerabilities in cancer

**DOI:** 10.3389/fonc.2023.1256769

**Published:** 2023-10-09

**Authors:** Ricardo A. León-Letelier, Rongzhang Dou, Jody Vykoukal, Ali Hussein Abdel Sater, Edwin Ostrin, Samir Hanash, Johannes F. Fahrmann

**Affiliations:** ^1^ Department of Clinical Cancer Prevention, The University of Texas MD Anderson Cancer Center, Houston, TX, United States; ^2^ Department of General Internal Medicine, The University of Texas MD Anderson Cancer Center, Houston, TX, United States

**Keywords:** kynurenine pathway (KP), cancer, metabolism, therapeutics, immune evasion

## Abstract

The kynurenine pathway (KP) and associated catabolites play key roles in promoting tumor progression and modulating the host anti-tumor immune response. To date, considerable focus has been on the role of indoleamine 2,3-dioxygenase 1 (IDO1) and its catabolite, kynurenine (Kyn). However, increasing evidence has demonstrated that downstream KP enzymes and their associated metabolite products can also elicit tumor-microenvironment immune suppression. These advancements in our understanding of the tumor promotive role of the KP have led to the conception of novel therapeutic strategies to target the KP pathway for anti-cancer effects and reversal of immune escape. This review aims to 1) highlight the known biological functions of key enzymes in the KP, and 2) provide a comprehensive overview of existing and emerging therapies aimed at targeting discrete enzymes in the KP for anti-cancer treatment.

## 1 Introduction

The kynurenine pathway (KP) has been recognized as a key mediator of tumor immune escape (1–3). Three enzymes, indolamine 2,3-dioxygenase 1 and 2 (IDO1, IDO2) and tryptophan 2,3-dioxygenase (TDO) initiate the first steps in the KP, converting tryptophan (Trp) to N-formyl-kynurenine, which can be further metabolized by arylformamidase (AFMID) yielding the immunosuppressive catabolite kynurenine (Kyn). Whereas TDO is predominately expressed in the liver and accounts for the majority of Trp metabolism, IDO-mediated metabolism predominately occurs secondary to inflammation (4, 5). Upregulation of IDO in tumor cells or antigen-presenting cells leads to Trp depletion and accumulation of its downstream catabolite Kyn in the local tumor microenvironment (TME), resulting in immunosuppression by inducing T cell anergy and apoptosis and suppressing T cell differentiation (1–3, 6–9). Building upon this underlying biological mechanism, substantial efforts have been dedicated to the development of small molecule inhibitors and agonists targeting the KP as anti-cancer agents to circumvent tumor immune suppression. Among the most investigated IDO inhibitors are epacadostat, navoximod, and indoximod, each of which have been explored as a monotherapy and in combination with immune check-point inhibitors (ICI) and/or chemotherapy (10–21). Despite early success in Phase I/II clinical trials, ECHO-202/KEYNOTE-037 failed to demonstrate additional benefit of epacadostat in combination with pembrolizumab (10) and similar findings have since been reported for navoximod with atezolizumab (19), raising uncertainty about the benefits of targeting IDO1 for anti-cancer treatment.

Yet, it is increasingly recognized that, beyond the well covered Trp-IDO-Kyn axis, other enzymes in the KP along with their associated catabolites can modulate the TME to evade immune surveillance and enable tumor progression (22–26). Moreover, emerging data suggests that KP-related enzymes, such as IDO1, can promote tumor progression through functions that are independent of enzymatic activity (27). The purpose of this review is to 1) highlight the known tumor promotive functions of key enzymes in the KP and 2) provide a comprehensive overview of existing and emerging therapies aimed at targeting discrete enzymes in the KP for anti-cancer treatment.

## 2 Role of the kynurenine pathway in cancer development and immune suppression

### 2.1 Indoleamine 2,3-dioxygenase 1

**Figure 1:**
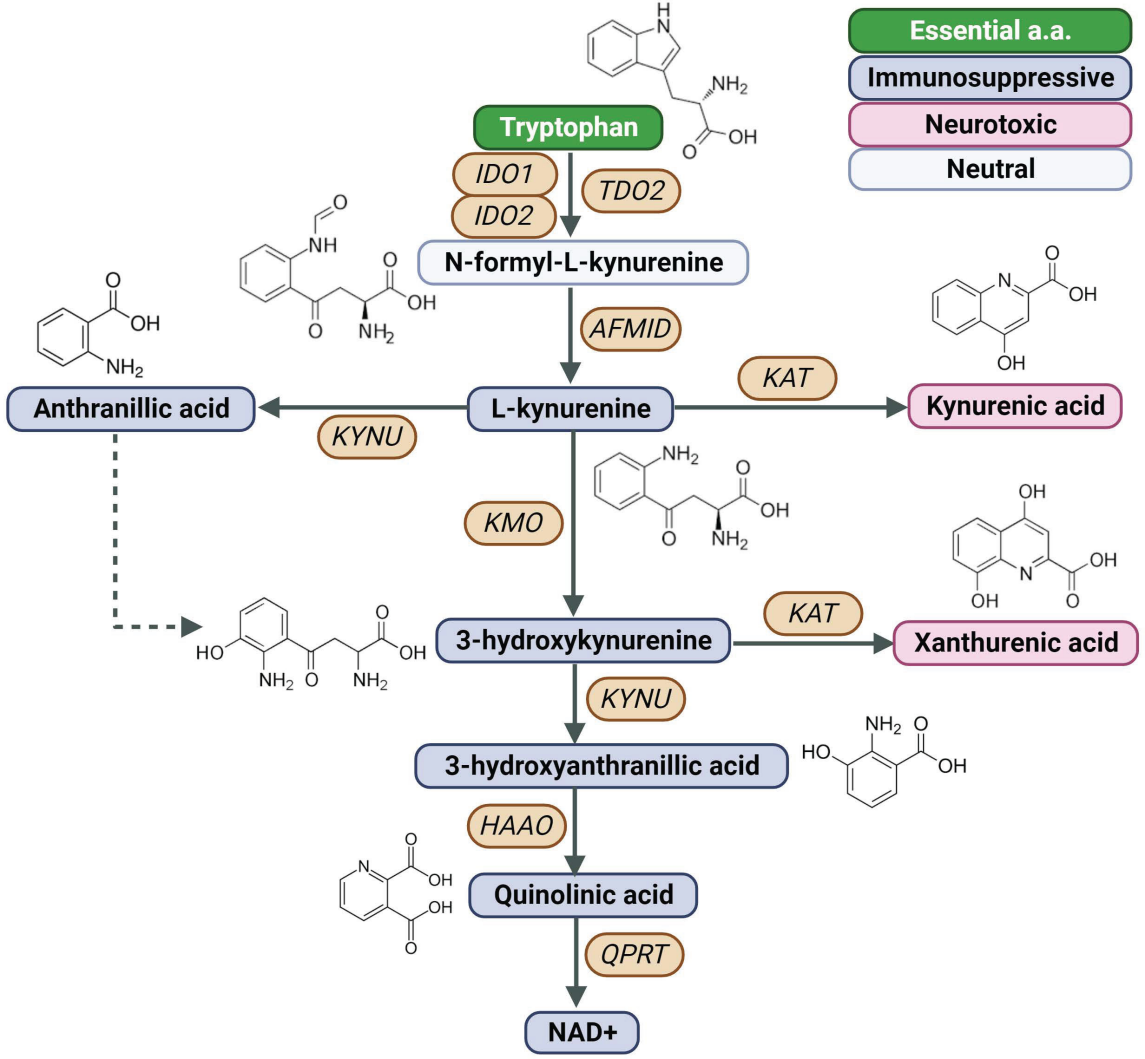
Created with BioRender.com. Schematic of the KP, Kynurenine Pathway; AFMID, arylformamidase; HAAO, 3-hydroxyanthranilate 3,4-dioxygenase; IDO1, indoleamine 2,3-dioxygenase 1; IDO2, indoleamine 2,3-dioxygenase 2; KAT, kynurenine aminotransferase; KMO, kynurenine 3-monooxygenase; KYNU, kynureninase; NAD+, nicotinamide adenine dinucleotide; TDO2, tryptophan 2,3-dioxygenase; QPTR, quinolinic acid phosphoribosyl transferase.

Indoleamine 2,3-dioxygenase 1 (IDO1) is a rate-limiting enzyme in the metabolism of the essential amino acid Trp into downstream kynurenines (**Figure 1**) (7). Under physiological conditions, the IDO1 functions to generate cellular nicotinamide adenine dinucleotide (NAD+). However, IDO1 is overexpressed in several cancer types (28, 29) and is prognostic for poor disease-free survival and overall survival (30, 31).

Seminal studies by Munn and colleagues demonstrated that reduced bioavailability of Trp due to IDO overactivation in dendritic cells (DCs) leads to accumulation of uncharged Trp-tRNAs in T cells and that the accumulation of Trp-tRNAs induces integrated stress response kinase (GCN2)-mediated suppression of the translation initiation factor 2a (eIF2a) resulting in subsequent cell growth arrest (6). Tryptophan starvation-mediated activation of GCN2 has also been shown to down-regulate the T cell receptor (TCR) zeta-chain in naïve T cells, resulting in diminished T cell function (32). Another study demonstrated a GCN2-independent mechanism whereby IDO-mediated catabolism of Trp inhibits immunoregulatory kinases mTOR and PKC resulting in induction of autophagy and immunosuppression (18). Subsequent studies demonstrated that IDO-mediated catabolism of Trp results in the accumulation of Kyn (7) that stimulates ligand-activated transcription factor aryl hydrocarbon receptor (AhR) signaling and promotes the generation of immune-tolerant DCs and regulator T cells (Tregs) (1, 7, 8). To this end, Liu and colleagues demonstrated that interferon-gamma (IFNγ) produced by cytotoxic CD8+ T cells stimulates Kyn release by tumor-repopulating cells (TRCs) and that TRC-derived Kyn is subsequently taken up by CD8+ T cells through TCR-mediated upregulation of solute carrier family 7 member 8 (SLC7A8) and Solute Carrier Family 36 Member 4 (SLC36A4, also known as PAT4), resulting in Kyn-AhR-mediated upregulation of programmed cell death protein 1 (PD-1) (33). Inhibition of IDO1 in CD8+ T cells promoted Trp accumulation and downregulation of PD-1 (34). Another study found that IFNγ secreted by acute myeloid leukemia (AML) cells upregulated IFNγ-dependent genes related to Treg induction, including IDO1, in mesenchymal stromal cells (MSCs). Genetic ablation of IFNγ production by AML cells reduced MSC IDO1 expression and Treg infiltration, thereby attenuating AML engraftment (35).

The IDO1-Kyn-AhR axis has also been shown to impair natural killer (NK) cell and macrophage function. Specifically, Kyn inhibits expression of the natural cytotoxicity triggering receptor 1 (NCR1, also known as NKp46) and killer cell lectin like receptor K1 (KLRK1, also referred to as NKG2D) on NK cells resulting in impaired tumor-killing functions (36, 37). Recently, Fang and colleagues demonstrated that IDO1 regulates expression of ADAM metallopeptidase domain 10 (ADAM10) via IDO1-Kyn-AhR pathway in non-small cell lung cancer (NSCLC) cells and that the induction of ADAM10 downregulates the NKG2D Ligand (NKG2DL). Targeting of IDO1 via RY103, a novel IDO1 inhibitor, improved NK cell-mediate anti-tumor activity in xenograft mouse models of lung cancer (38). Takenaka and colleagues reported that increased IDO-mediated release of Kyn by glioblastoma cells promotes AhR activation in tumor-associated macrophages (TAMs); activation of AhR signaling in TAMs increased expression of the ectonucleotidase CD39 and accumulation of adenosine in the tumor microenvironment (TME) leading to suppression of CD8+ T cell function (39). Interestingly, studies by Campesato and colleagues reported that IDO-Kyn-AhR-mediated immunosuppression depends on the interplay between Tregs and TAMs (40). Using a preclinical mouse model of B16-F10 melanoma overexpressing IDO1 (B16^IDO^), authors demonstrated that IDO-expressing tumors have marked increases in Treg frequency and upregulated expression of FoxP3 in tumor infiltrating CD4+ T cells compared to B16^WT^-tumor bearing mice. Concomitant to Tregs was an increased abundance of TAMs (CD11b+F4/80highLy6G−) that accumulated during tumor progression. Gene expression analyses of B16^IDO^-derived Tregs and TAMs revealed prominent upregulation of AhR and AhR-responsive genes. Depletion of macrophages with αCSF1-R or clodronate liposomes delayed the progression of IDO-expressing tumors but not wild-type tumors, an effect that could be reversed upon CD8+ T cell depletion. Similarly, specific depletion of Tregs using Foxp3DTR mice significantly abrogated the myeloid-enriched phenotype found in B16^IDO^ tumors, with reduced infiltration of CD11b+ cells and M2-like TAMs after treatment with DT. Co-culture experiments further demonstrated that Treg inhibitor function and expansion of M2-like macrophages was dependent on Kyn-AhR signaling (40).

Whereas IDO1 has largely been studied in the context of Trp-IDO1-Kyn-AhR signaling, IDO1 may also promote tumor progression through non-enzymatic functions (27). To this end, IDO1 can act as a signal-transducing molecule via two immunoreceptor Tyrosine-based inhibitory motifs (ITIMs) in the non-enzymatic domain of the enzyme. Phosphorylation of the ITIMs promotes interaction with SHP family tyrosine phosphatases (41, 42). Moreover, IDO1 can directly bind class IA PI3K regulatory subunits, resulting in IDO1 anchoring to early endosomes and activation of ITIM-mediated signaling (43).

In the context of cancer, using B16-F10 mouse melanoma cells transfected with either the wild-type *Ido1* gene (*Ido1^WT^*) or a mutated variant lacking the catalytic, but not signaling activity (*Ido1^H350A^*), Orecchini and colleagues demonstrated that IDO1 promote tumor growth by increasing SHP-2-mediated activation of Ras and Erk signaling *in vitro* and *in vivo*. Furthermore, B16^H350A^ tumor bearing mice exhibited markedly enhanced tumor growth, and worse overall survival compared to B16^wt^ or B16^mock^ control mice. Immunophenotyping of tumors indicated that B16^H350A^ tumors had lower IFNγ-producing CD4+ and CD8+ T lymphocytes and a significantly higher percentage of CD4^+^Fox3p^+^ Tregs compared to respective controls (27). Zhai and colleagues established IDO-deficient glioblastoma (GBM) cell lines reconstituted with IDO wild-type or IDO enzyme-null cDNA to assess tumor promotive roles of IDO1 independent of its catalytic activity. They found that nonenzymatic tumor cell IDO1 activity increased expression of complement factor H (CFH) and its isoform, factor H like protein (FHL-1) in human GBM, resulting in increased intratumoral Tregs and myeloid-derived suppressor cells, accelerated tumor growth and poor survival (44). These findings highlight an increasingly appreciated role of IDO1 in signal transduction, and, importantly, underscore an alternative mechanism of IDO1-mediated immune suppression that may not be impeded by small molecule IDO inhibitors.

### 2.2 Indoleamine 2,3-dioxygenase 2

**Figure 2:**
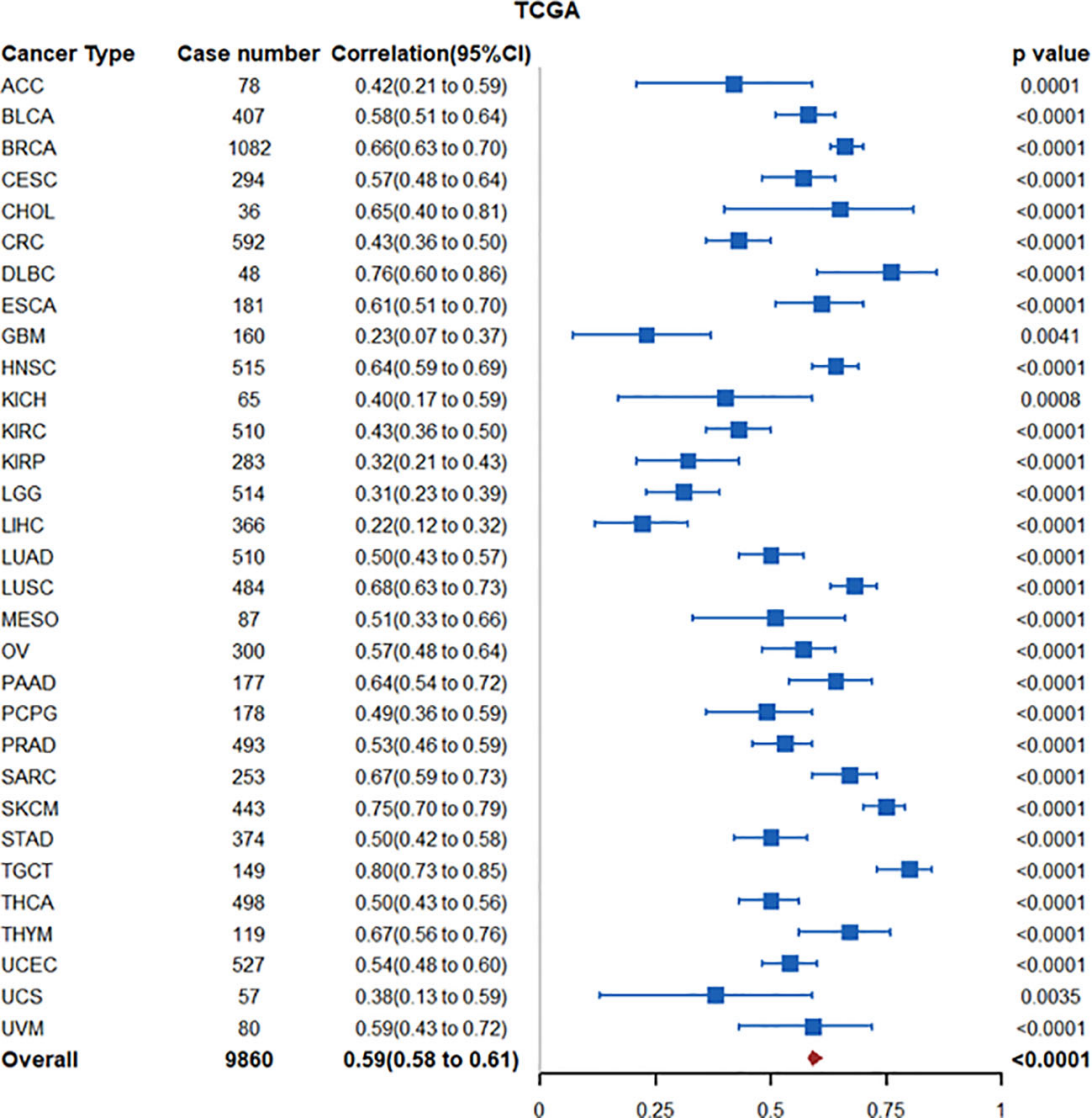
Forest plot illustrating association between IDO1 and IDO2 mRNA expression in the TCGA PanCancer Atlas transcriptomic datasets. Nodes represent spearman correlation coefficients, corresponding 95% confidence intervals, and 2-sided p-values.

The IDO1 paralogue IDO2 is another enzyme involved in the catabolism of tryptophan to downstream kynurenines. *INDOL1*, the encoding gene for IDO2, is located on chromosome 8p12 near *INDO* (located on chromosome 8p11), the encoding gene for IDO1 (45). In The Cancer Genome Atlas (TCGA)-PanCancer Atlas transcriptomic datasets, increased tumoral IDO2 is highly correlated with IDO1 expression (Spearman ρ coefficient: 0.59 (95% CI: 0.58-0.61)) (**Figure 2**), suggesting potential co-regulation (46). In this regard, studies by Kado and colleagues demonstrated that 2,3,7,8-tetrachlorodibenzio-p-dioxin (TCDD)-mediated activation of AhR signaling increased IDO2 expression in wildtype MCF7 breast cancer cells but not in CRISPR-cas9 AhR-knockout MCF-7 cells. Promoter analyses identified short-tandem repeat containing four core sequences of a xenobiotic response element (XRE) upstream of the start site of the human *ido2* gene, reinforcing AhR as an upstream regulator (47). TCDD-mediated AhR activation in DC cells also induced expression of IDO1 and IDO2 (48). Interestingly, Li and colleagues demonstrated that tolerogenic phenotype of IFN-γ-induced IDO2 expression in DCs is maintained via autocrine IDO-Kyn/AhR-IDO loop (49). Thus, in tumors with high IDO1/IDO2 expression, increased IDO2 expression may be driven through the IDO1-Kyn/AhR signaling axis.

With respect to cancer cell functions, small interfering RNA (siRNA)-mediated knockdown of IDO2 inhibited cancer cell proliferation, induced cell cycle arrest in G1 and apoptosis, and reduced cell migration of B16-BL6 melanoma cells *in vitro*. Mechanistically, IDO2 knockdown increased generation of reactive oxygen species (ROS), and decreased generation of NAD+ (50).

In the context of the tumor immunophenotype, a pan-cancer analysis of IDO2 in the TCGA transcriptomic datasets revealed IDO2 mRNA expression to be strongly associated with high infiltration of immune cells in the tumor microenvironment, as well as tumor mutational load (TMB), microsatellite instability (MSI), mismatch repair (MMR), and immune checkpoint related genes (51).

IDO2 may promote tumor immune suppression through the Trp-Kyn-AhR axis. Studies by Yamasuge and colleagues demonstrated that Ido2 knockout (KO) tumors had higher intratumor Trp and reduced Kyn levels compared to wild-type Ido2 tumors in a Lewis lung carcinoma mouse model, indicating Ido2 Trp catalytic activity (52). Moreover, Ido2 KO tumors had significantly higher CD3+ and CD8+ TILs compared to wild-type Ido2 tumors (52).

Yet, it is unlikely that the biological function(s) of IDO2 are completely redundant with that of IDO1. This is reinforced by the fact that the K_m_ of IDO2 for Trp is markedly lower than IDO1 (K_m_ 500- to 1000-fold lower), despite both enzymes containing residues necessary for Trp catalytic activity (53, 54). Indeed, studies in autoimmune disease have identified several functions of IDO2 that are independent of IDO1 and Trp catalytic activity. For instance, Metz and colleagues found that IDO1-dependent T regulatory cell generation is defective in *Ido2^-/-^* mice. Mechanistically, *Ido2* deficient mice, but not wild-type *Ido2* mice, exhibited lower expression of immune regulatory cytokines, including IFN-γ, TNF-α, IL-6, and MCP-1/CCL2 (55). Merlo and colleagues reported that IDO1 mediates T cell suppressive effects whereas IDO2 mediates autoreactive B cell responses to promote inflammation (56). Using a catalytically inactive IDO2 knock-in mouse model, authors demonstrated that IDO2, but not IDO1, can directly interact with GAPDH, Runx1, RANbp10, and Mgea5 to potentiate an inflammatory response in autoimmune arthritis (57).

The above studies lend to an intriguing question as to whether a concomitant upregulation of tumoral IDO1 and IDO2 may synergize to suppress an anti-cancer immune response, which may lend to additional opportunities for cancer interception (*see Section 2 below*).

### 2.3 Tryptophan 2,3-dioxygenase

Liver-associated tryptophan 2,3-dioxygenase (TDO2) is another heme-containing enzyme that is involved in the catabolism of Trp to downstream kynurenines (**Figure 1**) and that has been reported to be frequently overexpressed in various malignancies (58). Elevated tumoral TDO2 expression is associated with poor prognosis in several cancer types including kidney renal papillary cell carcinoma, glioma, testicular germ cell tumors, and uveal melanoma (58) as well as liver (59), colorectal (CRC) (60), and breast cancer (61). Moreover, high TDO2 expression level positively correlated with higher immune infiltration, especially DCs, as well as immune checkpoint-related gene markers, such as LAIR1, CD276, NRP1, CD80, and CD86 (58). Regulation of TDO2 expression has been linked to IL-1β- C/EBPβ-MAPK signaling activities (62).

Like IDO1, TDO2-mediated Kyn promotes tumor immune suppression through AhR-signaling in various cancer types (63, 64). To this end, single-cell RNA sequencing (scRNA-seq) of 13 cancerous tissues identified a subset of myofibroblasts that exclusively expressed TDO2 and that clustered with CD4+ and CD8+ T cells distal to tumor nests. Functional experiments demonstrated that TDO2+ myofibroblasts induce transformation of CD4+ T cells into Tregs and caused CD8+ T cell dysfunction. In a murine model of oral squamous cell carcinoma (OSCC), small molecule inhibition of TDO2 via LM10 attenuated the inhibitory states of T cells, restored the T cell antitumor response, and prevented malignant transformation (65). Interestingly, Schramme and colleagues demonstrated that C57BL/6 TDO-KO mice (66) engrafted with MC38 CRC cells were more sensitive to anti-CTLA4 or anti-PD1 treatment compared to MC38-tumor bearing wild-type C57BL/6 mice. Enhanced efficacy of ICI in C57BL/6 TDO-KO tumor bearing mice was attributed to higher systemic Trp levels, which could be reversed through a Trp-low diet (67). These studies implicate host metabolism Trp independent of cancer TDO2 status as being a determinant of response to ICI.

In addition to its role in immune suppression, TDO2 has also been demonstrated to promote migration and invasion of liver cancer cells via Wnt5a signaling activities (59). Another study demonstrated that TDO2-Kyn-AhR activation in liver cancer cells induces autocrine interleukin 6 (IL-6)-mediated Signal transducer and activator of transcription 3 (STAT3) and Nuclear factor kappa beta (NFκB)/T Cell Immunoglobulin And Mucin Domain Containing 4 (TIM4) signaling to promote tumor progression (68). Inhibition of AhR signaling by PDM2 attenuated tumor growth in a xenograft model of liver cancer (68). In the context of CRC, deficiency in adenomatous polyposis coli (APC) results in TCF4/β-catenin-mediated upregulation of the TDO2-Kyn-AhR axis to increase glycolysis and drive anabolic cancer cell growth (69). Knockdown of TDO2 in LoVo and HCT116 CRC cells attenuated cell growth, and reduced migration, invasion and colony formation potential through inactivation of TDO2-kynureninase (KYNU)-AhR signaling (60).

### 2.4 Arylformamidase

Arylformamidase (AFMID) is another downstream KP enzyme that is frequently overexpressed in various malignancies (70), and that catalyzes the hydrolysis of N-formyl-Kynurenine into Kyn and formate (**Figure 1**) (71). Although the functional role of AFMID upregulation in cancer remains poorly understood, Venkateswaran and colleagues demonstrated that oncogenic MYC increases expression of the Trp importers SLC1A5 and SLC7A5 as well as AFMID in colon cancer. Increased uptake and catabolism of Trp results in the accumulation of Kyn that enables cancer cell proliferation in part through AhR-signaling (72).

Splicing changes in *AFMID* have also been associated with survival and relapse in patients with hepatocellular carcinoma (HCC) (73). Specifically, the switch of AFMID isoforms was found to be an early event in HCC development and was associated with driver mutations in *TP53* and AT-rich interactive domain-containing protein 1A (*ARIDIA).* Authors further found that overexpression of the full-length AFMID isoform leads to higher NAD+ levels, lower DNA-damage response, and slower cell growth in HepG2 cells (73). To this end, Tummala and colleagues found that loss of AFMID expression in SNU-449 HCC cells resulted depletion of NAD+, indicating that Trp catabolism in HCC cells supports *de novo* NAD+ synthesis (74). Additional studies are warranted to better elucidate the functional relevance of AFMID in tumorigenesis and progression; however, one may surmise that the elevation of AFMID underlies increased accumulation and secretion of Kyn.

### 2.5 Kynureninase

Kynureninase (KYNU) is a pyridoxal phosphate (PLP)-dependent hydrolase that catalyzes the cleavage of Kyn as well as 3-hydroxykynurenine (3HK) into anthranilic acid (AA) and 3-hydroxyanthranilic acid (3HA), respectively (**Figure 1**) (75). KYNU has been strongly implicated in the inflammation and immune modulation; however, the exact underlying mechanism(s) remain incomplete (76–81).

Recently, our group demonstrated selective enrichment of KYNU, but not other downstream KP enzymes, in lung adenocarcinomas (LUAD) harboring a loss-of-function *KEAP1* mutation (22). Metabolomic analyses confirmed that KYNU was enzymatically functional, as evidenced by the accumulation of AA in conditioned medium of LUAD cell lines. Activation of the Nuclear factor erythroid 2-related factor 2 (NRF2) pathway through siRNA-mediated knockdown of *KEAP1* or chemical induction with the NRF2-activator CDDO-Me upregulated KYNU at both the mRNA and protein levels with concomitant increases in AA production. Moreover, elevated tumoral KYNU expression was found to be associated with a tumor suppressive immunophenotype and was prognostic for poor overall survival in a tissue microarray (TMA) of LUAD as well as multiple independent LUAD transcriptomic datasets (22). A pan-cancer analyses further revealed upregulation of KYNU to be a prominent feature of NRF2-activated cancers and is associated with tumor immunosuppression and poor prognosis (23). Interestingly, a recent study by Liu and colleagues demonstrated that KYNY-derived 3HA has anti-oxidant properties and that elevations in cancer cell intracellular 3HA pools infer resistance to ferroptosis (82).

Studies by Heng and colleagues similarly found that KYNU was highly upregulated in HER2-enriched and triple-negative (TN) breast cancers (BrCa), leading to increased production of AA and 3HA (81). Interestingly, stimulation of TNBC cell lines with IFNγ resulted in a pronounced 286-fold increase in 3HA and higher serum levels of 3HA was able to distinguish TNBC from the other BrCa molecular subtypes (81). These findings are notable given that 3HA has been shown to enhance the percentage of Tregs, inhibit T-helper (Th)-1 and Th17 cells (24) and suppress antigen-independent proliferation of CD8+ T cells (83). To this end, a large-scale transcriptomic analysis of 2,994 BrCa tumors found a strong correlation between tumoral KYNU with inflammatory and immune responses; elevated KYNU was also linked to BrCa grade and poorer clinical outcomes (78).

In addition to cancer cell-associated upregulation of KYNU, a recent study by Itoh and colleagues found that KYNU is also elevated in cancer-associated fibroblast (CAF)-educated fibroblasts (CEFs). Specifically, authors demonstrated that CAFs induce CEF generation from normal fibroblasts (NFs) via reactive oxygen species (ROS)-induced activation of NFκB signaling, resulting in induction of a pro-inflammatory loop and secretion of asporin (ASPN), a small leucine-rich proteoglycan. ASPN in turn promoted upregulation of IDO1 and KYNU in CEFs, which induced cytocidal effects against CD8+ T-cells and promoted tumor spreading (79).

### 2.6 Kynurenine 3-monooxygenase

Kynurenine 3-monooxygenase (KMO) is a flavin adenine dinucleotide (FAD)-dependent monooxygenase and is located in the outer mitochondrial membrane that catalyzes the hydroxylation of Kyn into 3-hydroxykynurenine (3HK) (**Figure 1**) (84). Upregulation of KMO has been reported in several cancer types, including TNBC (85), CRC (86), HCC (87), and astrocytoma (88).

In the context of TNBC, elevated tumoral KMO is associated with worse overall survival and invasive breast cancers have among the highest rates of KMO copy number amplification (81, 89). *In vitro* ectopic overexpression of KMO in TNBC cell lines promoted increased cell growth, colony and mammosphere formation, migration and invasion, as well as increased expression of mesenchymal markers (85). In xenograft models, mice harboring CRISPR KMO-KD MDA-MB-231 TNBC cells had reduced tumor growth, attenuated capacity for lung metastases, and prolonged overall survival compared to respective control mice. Mechanistically, KMO prevented degradation of β-catenin, thereby enhancing the transcription of pluripotent genes, including CD44, Nanog, Oct4, and SOX2 (85). In support of the aforementioned finding, knockdown of KMO in CRC cells was also reported to decrease expression of cancer stem cell markers CD44 and Nanog, and reduce sphere formation as well as migration and invasion (86). Inhibition of KMO enzymatic activity, using the small molecule inhibitor UPF648, similarly attenuated sphere formation and cell motility of CRC cell lines (86). Interestingly, using immunohistochemistry, flow cytometry, immunofluorescence assay, and transmission electron microscopy, Lai and colleagues demonstrated that KMO is highly expressed on the cell membranes of breast cancer tissues and the cancer cell surfaceome of cell lines (90). Treatment of MDA-MB-231 TNBC cell lines with anti-KMO antibodies reduced the cell viability and inhibited the migration and invasion of the triple-negative (90). Collectively, these findings demonstrate a cancer cell intrinsic function of KMO that promotes tumor aggressiveness and pluripotency.

Notably, induction of KMO in immune cell subtypes has also been linked to dysfunctional anti-tumor activity. Using melanoma-derived cell lines and primary CD4+ CD25- T cell co-cultures, Rad Pour and colleagues demonstrated that activation of CD4+ T cells results in increased production of IFNγ with concomitant increases in Kyn and kynurenic acid that is attributed to reduced KMO expression in CD4+ T cells (25). Accumulation of Kyn and kynurenic acid suppresses CD4+ T cell expansion and viability (25). In the context of multiple myeloma (MM), Ray and colleagues demonstrated that KMO is upregulated in plasmacytoid dendritic cells (pDCs), resulting in immune suppression (26). Specifically, using co-culture models of patient autologous pDC–T–NK–MM cells, authors showed that pharmacological blockade of KMO activates pDCs and triggers both MM-specific cytotoxic T-cell lymphocytes (CTL) and NK cells` cytolytic activity against tumor cells. Combination treatment with R0-61-8048, a potent competitive inhibitor of KMO, and anti-PD-L1 antibody yielded superior anti-cancer efficacy compared to either treatment alone (26).

### 2.7 Kynurenine aminotransferase

Kynurenine aminotransferase family members (KATI-KATIV) catalyze the transamination of Kyn and 3HK into kynurenic acid (KA) (91) and xanthurenic acid (XANA), respectively (**Figure 1**) (4). Although limited information exists regarding the functional role of KAT in tumorigenesis, KAT-derived KA is readily detectable in tumor tissues, as well as in blood and urine of cancer patients (92).

Early studies demonstrated that KAT-derived KA exerts anti-proliferative effects by modulating key pathways associated with proliferation, survival, apoptosis, and migration (93–96). Yet, KA is documented to exert immunosuppressive effects via the G-protein-coupled receptor 35 (GPR35) (97), which may potentiate tumor progression (98). Moreover, KA has also been reported to be a potent agonist of the AhR that synergistically induces IL-6 (99) with potential pro-tumoral effects (100). To this end, a recent pan-tissue study revealed that interleukin-4-induced-1 (IL4I1) more frequently associates with AhR activity than does IDO1 or TDO2. Mechanistically IL4I1-mediated generation of indole metabolites and KAT-derived KA that activated AhR signaling to promote cancer cell motility and suppression of adoptive immunity (101). The above findings underscore the enigmatic role of KAT and KA in tumor progression. Further investigations are needed.

### 2.8 3-hydroxyanthranilate 3,4-dioxygenase

3-hydroxyanthranilate 3,4-dioxygenase (HAAO) is involved in the synthesis of quinolinic acid (QA) (**Figure 1**). Expression patterns of HAAO are variable among different cancer types (5, 22, 23); however, studies of endometrial carcinomas revealed promotor hypermethylation of HAAO is prominent and to be associated with microsatellite instability and poor clinical outcomes (102). Hypermethylation of HAAO has also been reported to be higher in prostate cancer tissues compared to adjacent control tissue, and it is associated with a more aggressive phenotype (103).

### 2.9 Quinolinic acid phosphoribosyl transferase

Quinolinic acid phosphoribosyl transferase (QPRT) is a rate-limiting enzyme in *de novo* NAD+ biosynthesis and catalyzes the conversion of QA to nicotinate ribonucleotide (**Figure 1**). The cancer cell intrinsic effects of QPRT upregulation have been studied in a variety of cancer types (104–109). For instance, neoplastic transformation in astrocytes is associated with a QPRT-mediated switch in NAD+ metabolism by exploiting microglia-derived quinolinic acid as an alternative source of replenishing intracellular NAD+ pools. Mechanistically, the increase in QPRT expression increases resistance to oxidative stress, enabling disease progression (104). Increased expression of QPRT in invasive breast cancer promotes cell migration and invasion through purinergic signaling (105). Another study found down syndrome cell adhesion molecule antisense RNA 1 (DSCAM-AS1) increases QPRT expression in breast cancer cells via competitively binding miRNA-150-5p and miRNA-2467-3p, resulting in increased cell growth, migration, and invasion of estrogen-receptor breast cancer cells (106). Upregulation of QPRT has also been shown to confer resistance to chemotherapy in leukemic cells and ovarian cancer (107, 108). In this regard, Thongon and colleagues evaluated the mechanisms of cancer cell resistance to nicotinamide phosphoribosyltransferase (NAMPT), the rate-limiting enzyme in NAD+ biosynthesis from nicotinamide. In their study, FK866-resistant CCRF-CEM (T cell acute lymphoblastic leukemia) cells were found to have exceptionally high QPRT activity and exhibited an addiction to exogenous Trp to maintain NAD+ pools under stress conditions (109).

Despite the above studies, QPRT has also been shown to be inversely associated with other KP pathway enzymes, suggesting reduced expression in some cancer types (5, 22, 23). For example, studies in renal cell carcinoma revealed QPRT to be downregulated in tumors and loss of QPRT expression led to anchorage-independent growth of RCC cells (110). Thus, the relevance of QPRT in promoting tumor progression is likely to be context dependent.

## 3 Targeting the kynurenine pathway for anti-cancer treatment

Based on intrinsic malignant properties as well as tumor immune suppression, targeting of the kynurenine pathway has garnered considerable attention, with several small molecule inhibitors being explored for the treatment of solid malignancies as monotherapies or in combination with immunotherapy (**Table 1**). The below sections highlight existing small molecule inhibitors as well as emergent therapies that target the KP pathway for anti-cancer treatment.

**Table 1:** Existing and emerging therapies that target the Kynurenine Pathway.

Compound	Structure	Cancer types	Phase
IDO1, IDO2 and TDO2 inhibitors			
**Epacadostat**	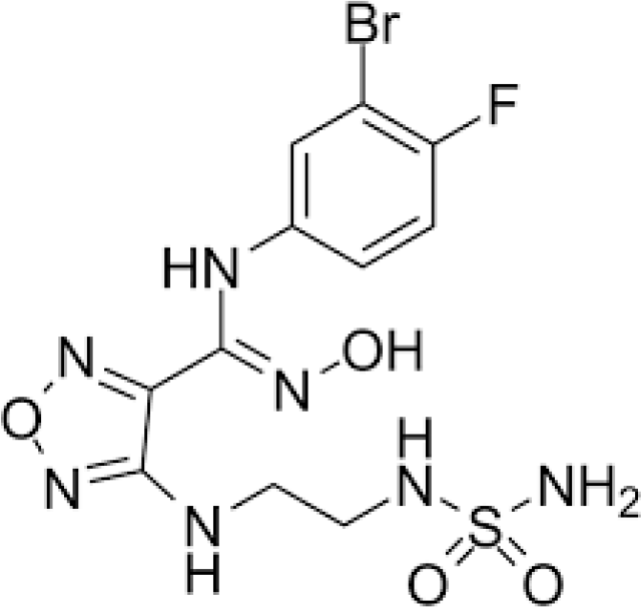	SKCM, RCC, NSCLC, BLCA, SARC, CRC, OVC	Clinical/Phase III
**Navoximod**	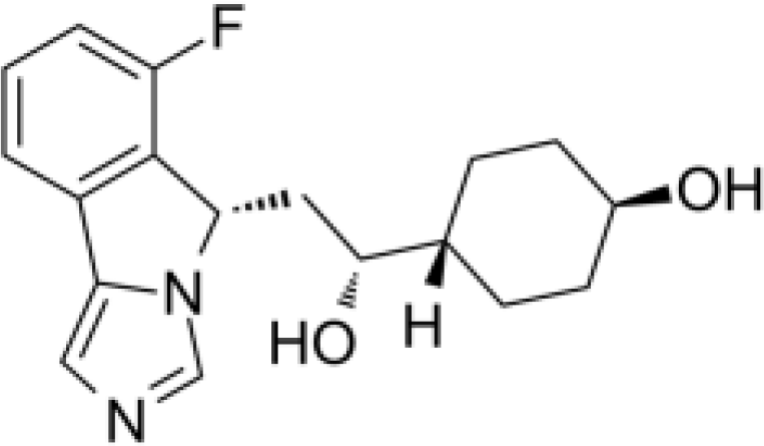	Solid tumors	Clinical/Phase I
**Indoximod**	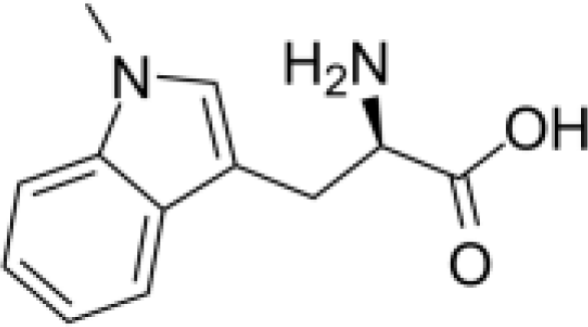	SKCM	Clinical/Phase II/III
**BMS-9862424**	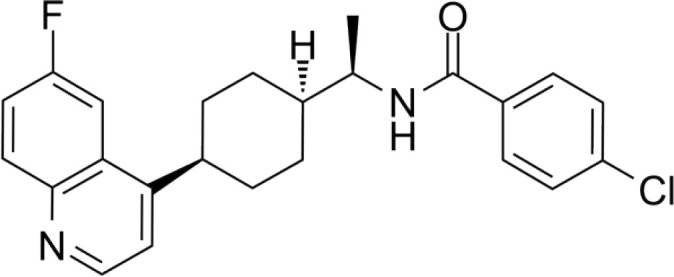	OVC	PD
**Linrodostat**	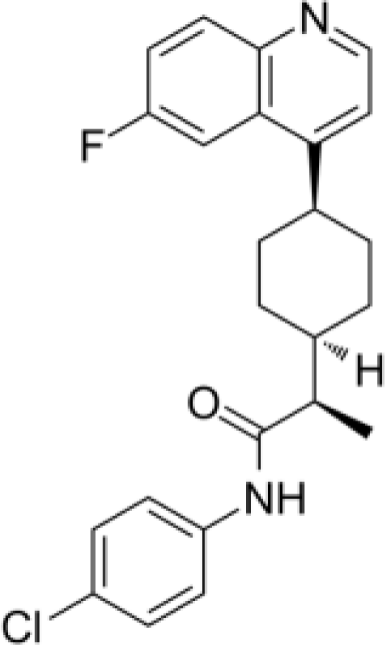	BLCA,	Clinical/Phase III
**YH29407**	No information available	CRC	Preclinical
**PF-06840003**	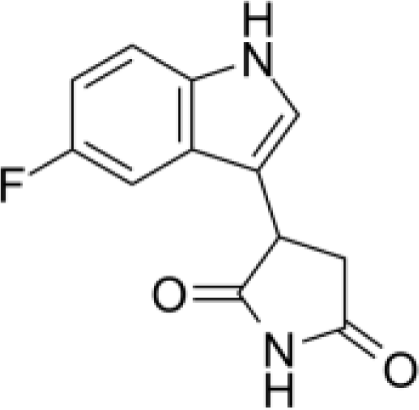	MG	Clinical/Phase I
**KHK2455**	No information available	Solid tumors	Clinical/Phase I
**LY3381916**	No information available	Solid tumors, NSCLC, RCC, TNBC	Clinical/Phase I
**MK-7162**	No information available	Solid tumors	Clinical/Phase I
**IACS-9779**	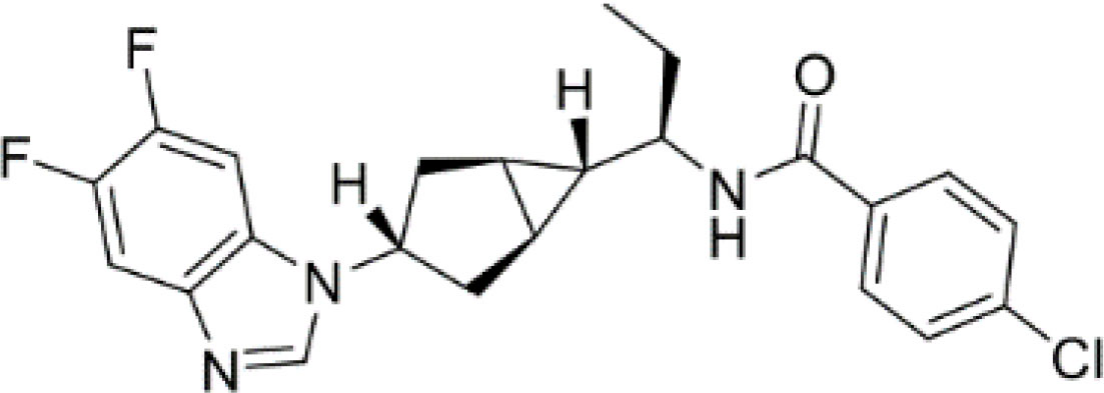	None	*in vitro* PD
**IACS-70465**	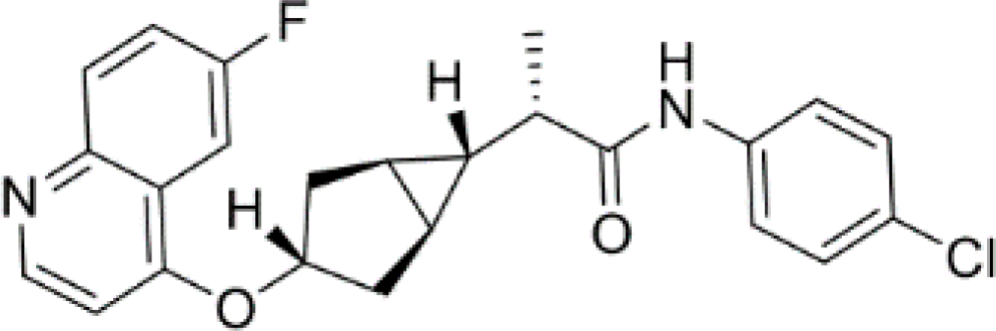	None	*in vitro* PD
**IDO1-PROTAC**	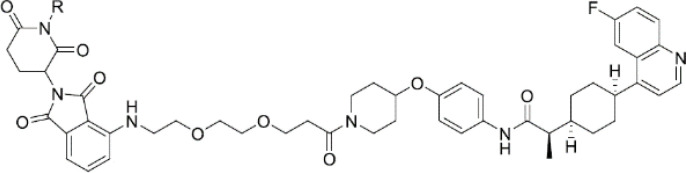	GBM	Preclinical PD
**IU1**	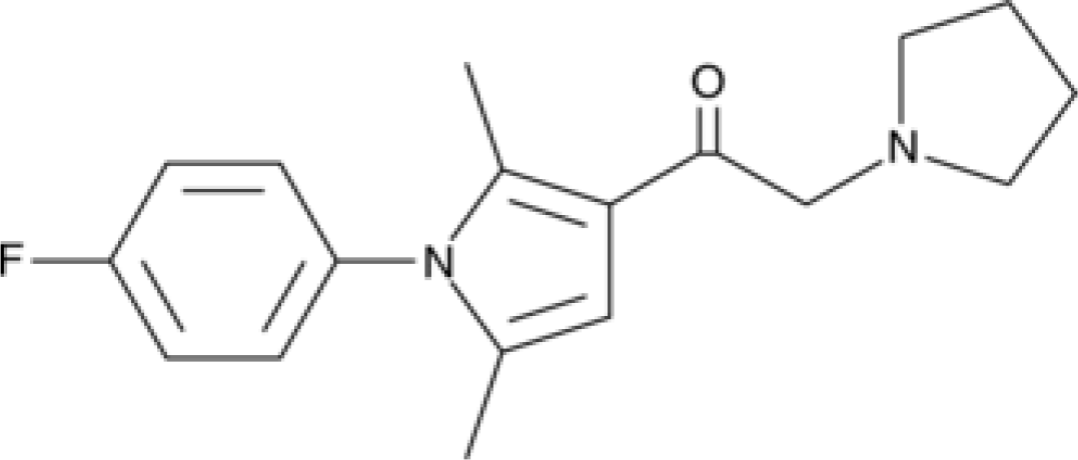	CRC	Preclinical
**3,4-dichloro aryl ring diaryl hydroxylamine**	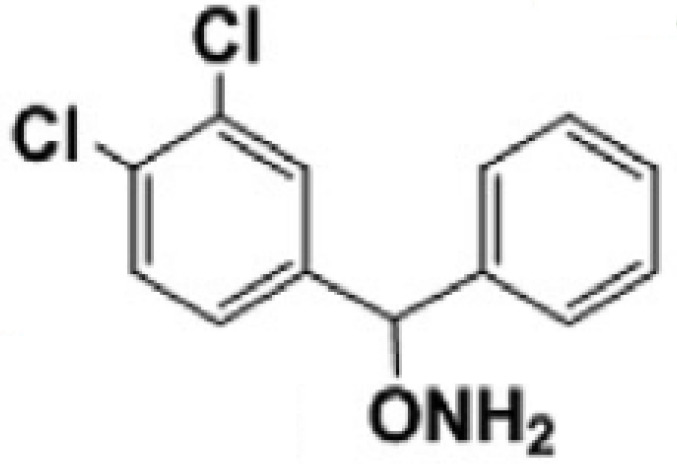	None	Preclinical PD
**2-chloro aryl ring diaryl hydroxylamine**	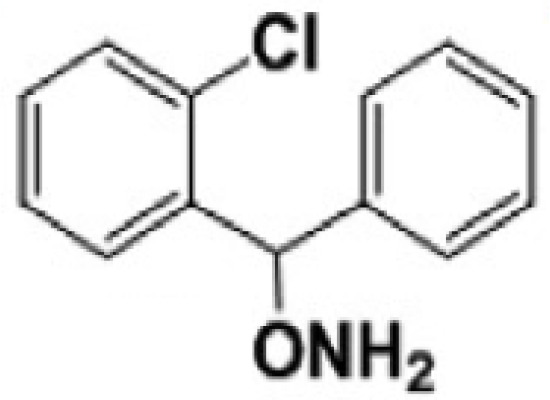	None	Preclinical PD
**4-aryl-1,2,3-triazole**	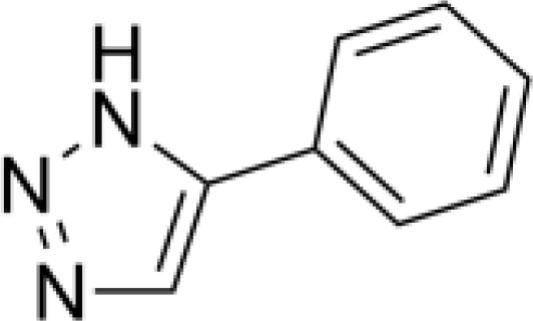	None	*in vitro* PD
**Tenatoprazole**	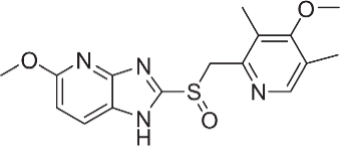	None	*in vitro*
**Compound 4t** **(He X.)**	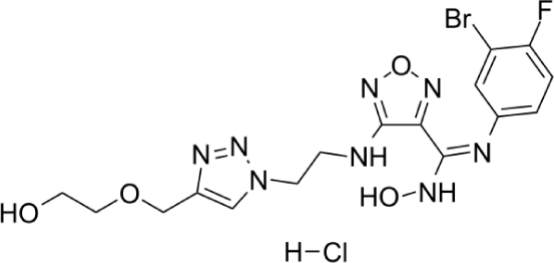	CRC	*in vivo*
**W-0019482**	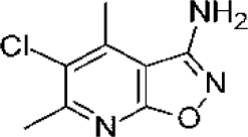	GBM, LLC	Preclinical
**680C91**	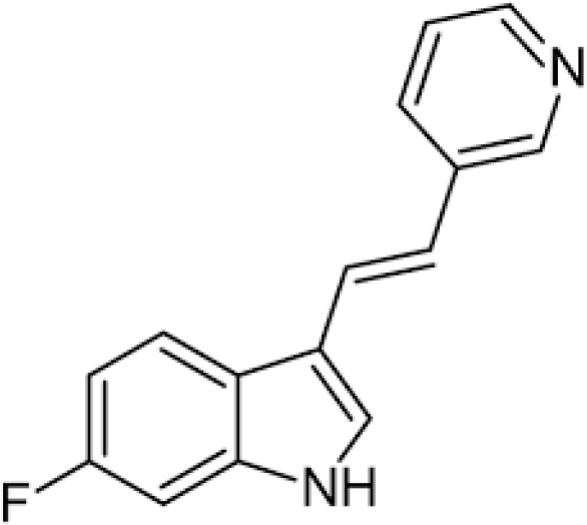	SKCM, CRC, LM, HCC	*in vitro*
**LM10**	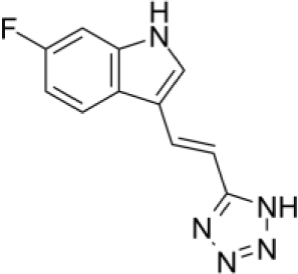	HCC	*in vitro*
**Sodium Tanshinone IIA Sulfonate**	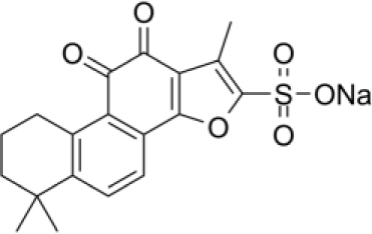	CRC	Preclinical
**RG70099**	No information available	GBM	Preclinical
**M4112**	No information available	Healthy subjects	Clinical/Phase I
**HTI-1090**	No information available	Solid tumors	Clinical/Phase I
**DN1406131**	No information available	Healthy subjects	Clinical/Phase I
**IACS-8968**	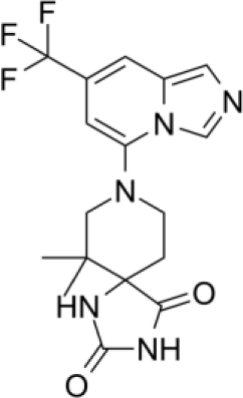	GBM	Preclinical
**EPL-1410**	No information available	SKCM, CRC	Preclinical
**RY103**	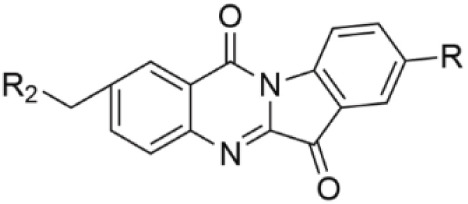	GBM, PC	Preclinical
**Compound 4** **(Hua S.)**	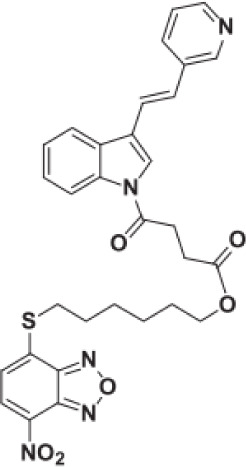	HCC	*in vitro*
**Compound 5** **(Hua S.)**	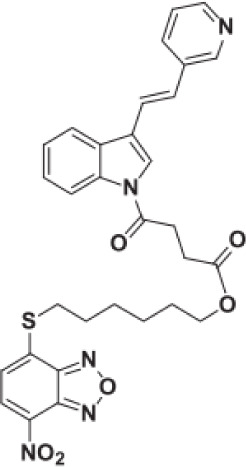	HCC	*in vitro*
KYNU inducers			
**S-phenyl-l-cysteine sulfoxide**	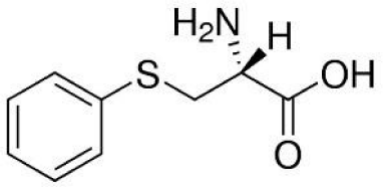	NA	*in vitro*
**Oestrone sulphate**	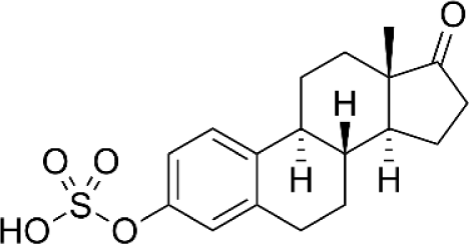	NA	*in vitro*
**O-methoxybenzoylalanine**	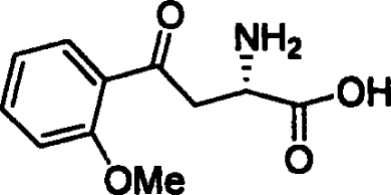	NA	*in vitro*
**Bensarazide hydrochloride**	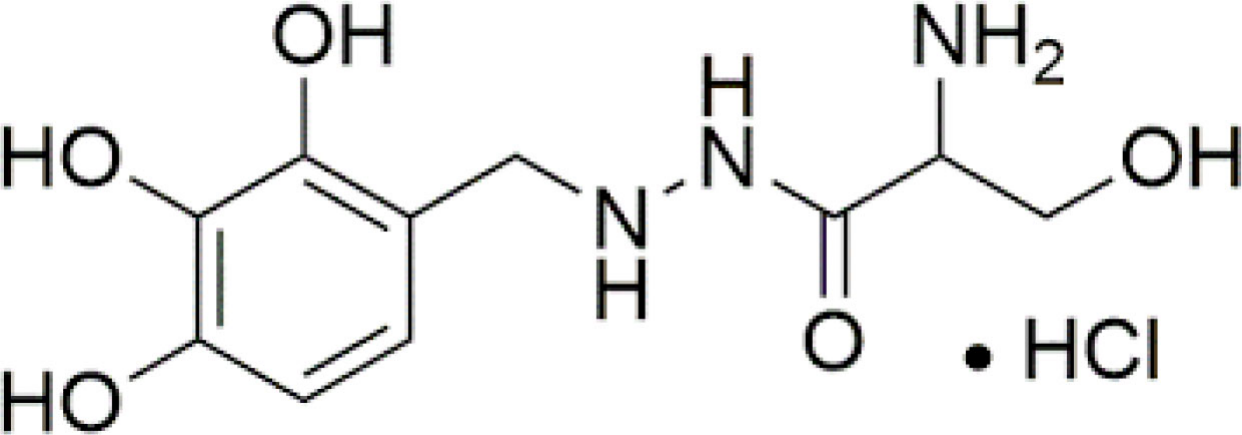	NA	*in vitro*
**PEGylated-KYNU**	NA	SKCM, TNBC, CRC	Preclinical
KMO inhibitors			
**GSK065**	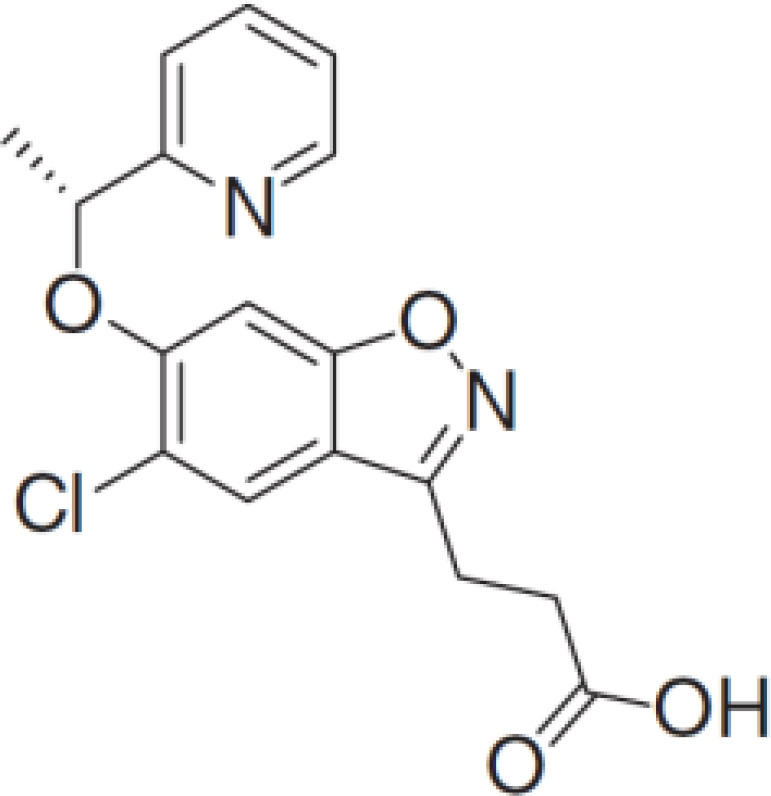	NA	*in vitro*
**GSK366**	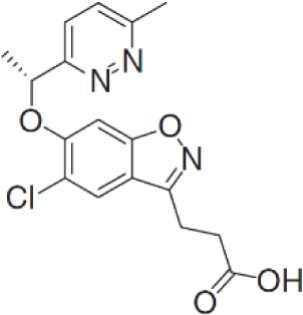	NA	*in vitro*
**UPF-648**	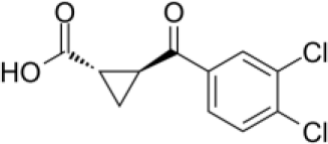	CRC	*in vitro*
**Ro 61-8048**	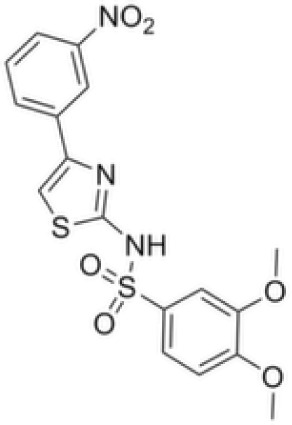	CRC	*in vitro*
**GSK3335065**	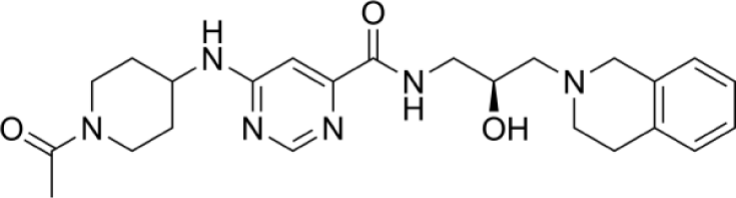	NA	Clinical/Phase I
HAAO inhibitors			
**NCR-631**	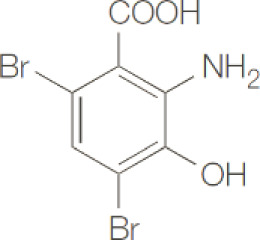	NA	Preclinical
**3-hydroxyanthranilic acid derivatives**	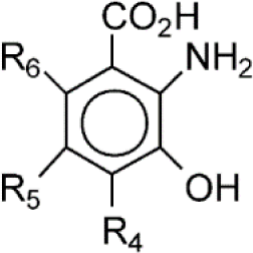	NA	Preclinical
QPRT inhibitors			
**Phthalic acid**	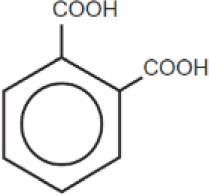	BRCA	*in vitro*
**NF340**	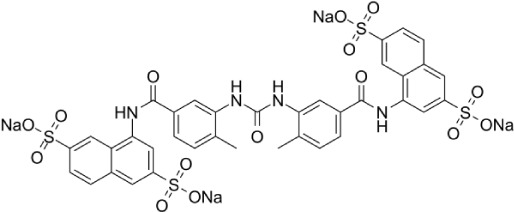	BRCA	*in vitro*

BLCA, bladder urothelial carcinoma; BRCA, breast cancer; CRC, colorectal cancer; GBM, glioblastoma; HCC, hepatocellular carcinoma; LALM, acute myeloid leukemia; LLC, Lewis lung carcinoma; LM, leiomyoma; MG, malignant glioma; NSCLC, non-small cell lung cancer; OVC, ovarian cancer; PD, pharmacodynamic; RCC, renal cell carcinoma; SARC, sarcoma; SKCM, skin cutaneous melanoma; TNBC, triple negative breast cancer.

### 3.1 Indoleamine 2,3-dioxygenase 1

#### 3.1.1 Epacadostat

Epacadostat (INCB024360) is a selective competitive inhibitor of IDO1 that has been explored for the treatment of solid malignancies as both a standalone agent and in combination with immune check-point inhibitor (ICI) therapies (11–16).

Pharmacokinetic and pharmacodynamic studies have demonstrated that, as a monotherapy, epacadostat is generally well tolerated, with a maximal inhibition of IDO1 activity (based on reductions in circulating kynurenine levels) achieved at doses ≥ 100mg BID. Adverse events (AEs) associated with such treatment included fatigue, nausea, decreased appetite, vomiting, constipation, abdominal pain, diarrhea, dyspnea, back pain, and cough (11). In a Phase I clinical study evaluating efficacy of epacadostat in patients with advanced solid malignancies, 18 out of 52 patients achieved stable disease, with 7 patients (13.5%) having stable disease lasting ≥16 weeks (11).

A seminal study, ECHO-202/KEYNOTE-037, evaluated the efficacy of epacadostat in combination with the PD-1 inhibitor pembrolizumab in advanced melanoma, with reported objective response rates (ORR) of 56% and a disease control rates (DCR; complete response (CR) + partial response (PR) + stable disease (SD)) of 78% (111). Despite early success in Phase I and II clinical trials, a Phase III clinical trial (ECHO-301/KEYNOTE-252) did not demonstrate additional benefit of epacadostat + pembrolizumab compared to placebo + pembrolizumab alone in patients with unresectable or metastatic melanoma (10).

ECHO-202/KEYNOTE-037 also reported results from Phase I/II studies evaluating combination treatment of epacadostat + pembrolizumab in sixty-two patients with various advanced solid tumors (13). Antitumor activity was observed in several cancer types, with ORR being observed in 25 (40.3%) of 62 patients. Epacadostat 100 mg twice a day plus pembrolizumab 200 mg every 3 weeks was recommended for Phase II evaluation. In the Phase II study, an objective response (OR) occurred in 55% (12 of 22) patients with melanoma and in those with non–small-cell lung cancer, renal cell carcinoma, endometrial adenocarcinoma, urothelial carcinoma, and head and neck squamous cell carcinoma (13). For NSCLC, combination treatment of epacadostat + pembrolizumab resulted in a ORR (CR + PR) of 35% (14 out of 40 patients) and a DCR (CR+PR+SD) of 60% (24 out of 40 patients) (112). Similar results were reported for renal cell carcinoma (15) and urothelial carcinomas (16). ECHO-207/KEYNOTE-723, a Phase I/II study of epacadostat plus pembrolizumab and chemotherapy for advanced solid tumors, also demonstrated ORRs of 31.4% across all treatment groups (113). However, a Phase II study evaluating epacadostat + pembrolizumab in 30 patients with selective sarcoma subtypes failed to achieve meaningful antitumor activity, with ORRs of <5% (114).

Epacadostat has also been explored in combination with other ICIs, including ipilimuab, an anti-cytotoxic T-lymphocyte-associated protein 4 (CTLA4) antibody, and nivolumab, an anti-PD-1 antibody (14, 115). To this end, Gibney and colleagues reported the results of a Phase I/II study of epacadostat in combination with ipilimuab in patients with unresectable or metastatic melanoma (14). In this study, immunotherapy-naïve patients (n= 39) achieved an ORR of 26% according to the immune-related response criteria and 23% by the Response Evaluation Criteria in Solid Tumors (RECIST 1.1); no ORR was observed in patients who received prior immunotherapy (14). Combination treatment of epacadostat plus nivolumab in advanced solid tumors achieved respective DCR (CR+PR+SD) of 24%, 28%, and 100% for colorectal cancer, ovarian cancer, and melanoma (115).

#### 3.1.2 Navoximod

Navoximod is an orally available non-competitive inhibitor of IDO1 (17). An open-label Phase Ia study by Nayak-Kapoor and colleagues assessed the safety, pharmacokinetics, pharmacodynamics, and preliminary anti-tumor activity of navoximod as a monotherapy in 22 patients with recurrent/advanced solid tumors (116). Trial-associated adverse events, regardless of causality, included fatigue (59%), cough, decreased appetite, and pruritus (41% each), nausea (36%), and vomiting (27%). Grade ≥3 AEs occurred in 14 of the 22 patients; however, only 2 were related to navoximod. Navoximod was found to be rapidly absorbed (Tmax ~ 1 h) with a half-life of approximately 11 hours, and transiently decrease plasma kynurenine levels. No objective responses were met based on RECIST v1.1 criteria; however, stable disease was observed in 36% of efficacy-evaluable patients (116).

Combination of navoximod plus the anti-PD-L1 inhibitor atezolizumab has also been explored for anti-cancer treatment of solid malignancies (19–21). Results from an open-label Phase Ib trial of 52 patients treated with navoximod plus atezolizumab demonstrated acceptable toxicity profiles; efficacy data available for 45 patients included 4 (9%) patients with a partial response and 11 (24%) patients with stable disease (21). Another Phase I study of navoximod plus atezolizumab in 20 Japanese patients with advanced solid tumors achieved stable disease in 65% (13 out of 20) patients. No dose-limiting toxicities were observed. The recommended dose of navoximod monotherapy was determined as 1000 mg orally BID, and 1000 mg orally BID could be considered in combination with atezolizumab (20). Despite these findings, a larger Phase I study of navoximod plus atezolizumab in 157 patients with advanced solid tumors failed to show benefit of adding navoximod to atezolizumab (19).

#### 3.1.3 Indoximod

Indoximod, also known as 1-methyl tryptophan (1-MT), is an indirect inhibitor of IDO1/IDO2 and is reported to serve as a high-potency Trp mimetic that reverses mTORC1 inhibition and the accompanying autophagy that is induced by Trp depletion in cells (17, 18). Indoximod has been explored for anti-cancer treatment in several Phase I/II clinical trials, both as a monotherapy as well as in combination with chemotherapy (117–120).

Recently, Zakharia and colleagues reported results from a single-arm Phase II clinical trial evaluating the addition of indoximod to standard of care ICI (pembrolizumab, nivolumab, or ipilimumab) approved for melanoma (121). Indoximod was administered continuously (1200 mg orally two times per day), with concurrent ICI dosed per US Food and Drug Administration (FDA)-approved label. A total of 131 patients were enrolled; pembrolizumab was the most frequently used ICI (114 out of 131; 87%). Efficacy was evaluable in 89 patients from the Phase II cohort with non-ocular melanoma who received indoximod combined with pembrolizumab. The ORR for the evaluable population was 51%, with confirm CR of 20% and DCR of 70%. ORR were highest among PD-L1 positive patients (70% compared to 46% for PD-L1 negative patients). Median progression-free survival was 12.4 months (95% CI 6.4 to 24.9) (121). Another recently completed Phase I/II trial (NCT01042535) evaluated an adenovirus-p53 transduced DC vaccine together with indoximod for treatment of metastatic breast cancer. A total of 44 patients were recruited, of which 36 completed the study. The results of this trial are pending.

#### 3.1.4 Others IDO inhibitors

To date, several additional IDO inhibitors have been developed including BMS-986242 (122), linrodostat (BMS-986205) (123, 124), YH29407 (125), PF-06840003 (126–128), KHK2455 (129), LY3381916 (130), and MK-7162. To this end, a Phase I study of PF-06840003 in 17 patients with recurrent malignant glioma demonstrated acceptable toxicity profiles, and with a reported dose-limiting toxicity (DLT) rate of 500 mg BID (131). Disease control occurred in eight patients (47%). Mean duration of stable disease was 32.1 (12.1-72.3) weeks (131). ENERGIZE, a Phase III study of neoadjuvant chemotherapy alone or with nivolumab with or without linrodostat mesylate for muscle-invasive bladder cancer is currently recruiting (128). Phase I clinical trials for KHK2455,(NCT02867007) LY3381916,(NCT03343613- recently terminated) and MK-7162 (NCT03364049) are on-going.

In addition to the above inhibitors, structural elucidation studies have also led to the discovery of imidazopyridines as potent IDO1 inhibitors (132) and substituted oxalamides as novel heme-displacing IDO1 inhibitors (133), as well as the development of first in class IACS-9779 and IACS-70465 inhibitors that bind the IDO1 apoenzyme (134). Anti-cancer efficacy of the mentioned above agents has been demonstrated in preclinical models; however, they have not been explored yet in clinical trials.

Another alternative approach has been to degrade intratumor IDO1. Bollu and colleagues reported the results of a novel IDO1 degrader using proteolysis targeting chimera (PROTAC) technology, which utilizes an E3 ligase complex to ubiquitinate targets for proteasome-mediated degradation. The IDO1-PROTAC resulted in potent cereblon-mediated proteasomal degradation of IDO1, and prolonged survival in mice with established brain tumors (135). Interestingly, studies by Shi and colleagues suggest that IDO1 expression may be regulated post-transcriptionally. Specifically, authors found that overexpression of USP14, a proteosome-associated deubiquitinating enzyme, in CRC cells deubiquitinates the IDO1 at the K48 residue, thus preventing proteasomal degradation (136). Knockdown or pharmacological targeting of USP14 decreased IDO1 expression, independent of AhR signaling, and improved anti-tumor immunity in a MC38 orthotopic syngeneic mouse model of CRC. Combination treatment of IU1, a selective small molecule inhibitor of USP14, and anti-PD-1 resulted in improved anti-killing effects compared to either treatment alone (136). Targeting IDO1 protein stability, rather than enzymatic activity, may thus provide an alternative approach to circumvent IDO1-mediated anti-cancer immunity.

### 3.2 Indoleamine 2,3-dioxygenase 2 inhibitors

Although the exact crystal structure of IDO2 remains incomplete, its high homology with IDO1 has enabled development of IDO2 inhibitors, including several diaryl hydroxylamines and 1,2,3-triazoles. For example, diaryl hydroxylamine compounds with a 3,4-dichloro aryl ring substitution exerted potent pan (IDO1/IDO2/TDO) inhibitory function whereas a 2-chloro aryl ring substitution inhibited IDO1 and IDO2 activity with respective IC_50_ values of 18 and 25μM (137). In cellular assays, 4-Aryl-1,2,3-triazole derivatives were also found to inhibit IDO2 function, with the best compound having an IC_50_ value of 50mM for mouse IDO2, and a twofold higher selectivity over human IDO1. Functionally, the 4-Aryl-1,2,3-triazole compounds were found to occupy both the A and B pockets of the IDO active site, which inhibited IDO2 more strongly than IDO1. Notably, the μM activity of the 4-Aryl-1,2,3-triazole compounds were similar to L-1MT (138). Another screening study identified the proton pump inhibitor, tenatoprazole, to exhibit an IC_50_ value of 1.8μM for IDO2 with no observed inhibition of IDO1 or TDO. Similar findings were found with other proton pump inhibitors. Proton pump inhibitors, such as tenatoprazole, were predicted to have one heteroatom coordinating to the heme iron in the active site of IDO2 (139).

A more recent study by He and colleagues reported *in vitro* and *in vivo* findings of their novel dual IDO1/IDO2 inhibitor, 4t. Structurally, 4t is an 1,2,3-triazole with a hydroxyethyl ether substitution with excellent inhibitory activity (IC_50_ values of 28 nM and 144nM for IDO1 and IDO2, respectively). Functionally, the hydroxyethyl ether chain of 4t formed two hydrogen bonding interactions with Tyr244 and Lys366 of IDO2, contributing to improved activity against IDO2. Pharmacokinetic experiments demonstrated favorable profiles, with adequate membrane permeability, high plasma protein binding, and safety. Moreover, 4t exhibited potent anti-tumor activity in a CT26 colorectal xenograft mouse model (140). Although promising, it remains to be determined whether the above mentioned IDO2 inhibitors will yield satisfactory anti-tumor activity in the clinical setting.

### 3.3 Tryptophan 2,3-dioxygenase inhibitors

There has been a considerable interest in the development of small molecule inhibitors of TDO2 given recent evidence that TDO2 is upregulated in several cancer types and, like IDO, mediates the catabolism of Trp and promote tumor immune suppression (7). To-date, small molecule inhibitors with high affinity to TDO2 are limited (141, 142).

Recently, W-0019482 was identified to be a potent inhibitor of IDO1, resulting in pronounced reductions in plasma and intratumoral ratios of Kyn-to-Trp and delayed growth of subcutaneous GL261-hIDO1 tumors in mice. Synthetic modification of W-0019482 yielded several analogues with either dual or TDO2-selective profiles. Four analogues, SN36458, SN36896, SN36499, and SN36704, were found to be TDO2 selective, exhibiting IC50 values 5.8 to 8.1-fold lower than that measured against IDO1 (143). The utility of these newer generation TDO2 inhibitors for anti-cancer treatment remains to be determined. Studies using HepG2 liver cancer cells demonstrated that TDO2-inhibitors 680C91 or LM10 significantly reduced Trp degradation and that TDO2, but not IDO1, was the primary source of Kyn production in these cells (144). 680C91 has also been explored in other cancer types including melanoma (145–147), colon (147), leiomyomas (148), and gliomas (149).

Interestingly, Zhang and colleagues reported that sodium tanshinone IIA sulfonate (STS), a sulfonate derived from tanshinone IIA (TSN), reduced the enzymatic activities of IDO1 and TDO2 with a half inhibitory concentration (IC50) of less than 10 μM using enzymatic assays (150). STS markedly decreased Kyn synthesis in IDO1- or TDO2-overexpressing cell lines and reduced the percentage of Forkhead Box P3 (FOXP3) T cells. *In vivo*, STS delayed tumor growth and combination treatment of STS with anti-PD-1 yielded superior anti-cancer efficacy compared to either treatment alone (150).

With regards to dual IDO/TDO2 targeting therapies, preclinical studies demonstrated that RG70099, potent dual small molecule inhibitor (IC_50_ <100nM) (151), efficiently reduced Kyn levels in plasma by ~90%, and >95% in tumors and draining lymph nodes. Notably, compared to conventional IDO inhibitors, RG70099 was able to reduce tumor Kyn levels by >90% in a TDO+ tumor xenograft, reinforcing that RG70099 exerting TDO2 inhibitory activity (151). EPL-1410, a fused heterocycle-based analogue, showed IDO1/TDO2 inhibitor activity in biochemical assays and demonstrated a significant dose dependent pharmacological efficacy in reducing the tumor volume in the syngeneic cancer models of CT-26 colon carcinoma and B16F10 melanoma (152). Recently, a Phase I clinical trial of M4112, an oral small molecule dual inhibitor of IDO/TDO2, in 15 patients with advanced solid tumors reported tolerable toxicity profiles (153). Treatment-emergent adverse events included fatigue, nausea, and vomiting. Dose-limiting toxicities (DLTs) were observed in one patient (grade 3 allergic dermatitis), and one grade 2 QT prolongation that resulted in a dose reduction. Neither the maximum tolerated dose (MTD) nor recommended Phase II dose was achieved (153).

### 3.4 Kynurenine aminotransferase inhibitors

To date, there exist several selective inhibitors of KAT, however, their applications in the context of anti-cancer treatment has not been readily explored (154–156). Despite this, several cancer types overexpress KAT (5), and, as mentioned in the above sections, KAT-derived KYNA may serve as a ligand for AhR signaling, resulting in tumor immune suppression (101), Evaluation of KAT inhibitors for anti-cancer treatment and potential reversal of immune suppression warrants further exploration.

### 3.5 Kynureninase inhibitors and pegylated-Kynureninase

Considering recent findings by our group as well as others (22, 23, 78, 79, 81, 83, 157), targeting of KYNU for anti-cancer treatment and reversal of immune suppression warrants investigation. To date, a few kynurenine analogues have been developed that selectively inhibit KYNU enzymatic activity (158). Additional molecules reported to inhibit KYNU include S-phenyl-l-cysteine sulfoxide (159, 160), oestrone sulphate (161), O-methoxybenzoylalanine (162), and benserazide hydrochloride (163).

However, targeting of KYNU should be met with caution. KYNU mediates the catabolism of Kyn to AA, which is thought to be immunologically inert. Administration of PEGylated-KYNU in immune-competent preclinical models of melanoma, breast, and colon cancer resulted in drastic reductions in tumor KYN levels with concomitant elevations in KYNU-derived AA, resulting in increases in tumor-infiltrating CD8+ T cells and subsequent tumor reduction (8). Moreover, anti-cancer efficacy was further enhanced when combining PEG-KYNase with ICIs (8). Overexpression (OE) of KYNU in modified Chimeric Antigen Receptor (CAR)-T cells yields superior anti-cancer efficacy in a NALM6 acute lymphoblastic leukemia mouse model (164). Compared to KYNU-knockout (KO) CAR T cells, KYNU-OE CAR T cells had higher glucose uptake, increased proliferation in carboxifluorescein diacetate succinimidyl ester (CSFE) assays, and exhibited a higher percentage of effector memory cell and effector CAR T-cells. In cell killing assays, KYNU-OE CAR T cells had higher lytic granules of granzyme B and enhanced cytokine production of IFNγ, compared to KYNU-KO CAR T cells. Importantly, KYNU-OE CAR T cells retained anti-cancer efficacy in the presence of high KYN levels (164). In another study, an organic polymer nanoenzyme (SPNK) conjugated with kynureninase (KYNase) via PEGylated singlet oxygen (1O2) cleavable linker with near-infrared (NIR) photoactivatable immunotherapeutic effects was developed for photodynamic immunometabolic therapy (165). Upon NIR photoirradiation, SPNK generates 1O2 to induce the immunogenic cell death of cancer and released KYNase to degrade KYN in the TME. *In vivo*, SPNK treatment elicited effector T cell tumor infiltration and expansion, enhanced systemic antitumor T cell activity, and prolonged survival of tumor-bearing mice (165).

The potential of KYNU as a direct target of therapy or as the therapy itself (i.e. pegylated-KYNU) remains an active area of exploration.

### 3.6 Kynurenine 3-Monooxygenase inhibitors

To date, several inhibitors of KMO have been developed, the majority of which share a common pharmacophore containing both an acidic moiety and a mono or 1,2-dichloro substitution of the core phenyl ring (84, 166–169). More recent inhibitors, GSK065 and GSK366, trap the catalytic flavin in a previously unobserved tilting conformation, resulting in picomolar affinities, increased residence times, and an absence of the peroxide production observed with prior inhibitors (166).

Evaluation of KMO inhibitors for anti-cancer treatment remains limited; however, studies by Ray and colleagues demonstrated that Ro 61-8048, a potent, selective inhibitor of KMO (170), activates pDCs and enhances pDC-triggered T cell proliferation and enhanced anti-cancer activity (26). Specifically, in co-culture models of patient pDCs, T cells, or NK cells with autologous multiple myeloma (MM) cells, blockade of KMO via Ro 61-8048 resulted in a robust MM-specific CD8+ CTL activity as well as significantly increased NK cell cytolytic activity (26). Treatment of CRC cell lines with Ro 61-8048 or UPF 648, another KMO inhibitor, resulted in reduced viability, decreased sphere formation and attenuated cell migration and invasion (86).

The therapeutic potential of KMO inhibitors remains to be determined; however, a prior clinical trial of the KMO inhibitor GSK3335065 was conducted among healthy volunteers. While GSK3335065 rapidly increases kynurenine levels suggesting partial inhibition of KMO activity, the trial was terminated after a serious adverse event (SAE) occurred on the study and a relationship with the study drug could not be excluded (171).

### 3.7 3-hydroxyanthranilic acid 3,4-dioxygenase and Quinolinic acid phosphoribosyl transferase inhibitors

Presently, there are very few inhibitors available that selectively target HAAO. Existing HAAO inhibitors include NCR-631 (AstraZeneca, Sweden) (172) as well as 4,5-, 4,6-disubstituted and 4,5,6-trisubstituted 3-hydroxyanthranilic acid derivatives, which have been shown to reduce the production of the excitotoxin QA (173, 174). To the best of our knowledge, none of these inhibitors have been evaluated to anti-cancer treatment.

Regarding QPRT, existing small molecule inhibitors include phthalic acid (PA) as well as the P2Y11 antagonist (NF340) (105, 175, 176). To this end, phthalic acid and NF340 were able to attenuate QPRT-enhanced invasiveness pCMV6-QPRT-transfected MDA-MB-231 TNBC cell lines (105). Given that NAD is essential for metabolic and immune homeostasis, QPRT inhibitors for cancer therapy warrants investigation, although should be met with some caution due to potential off target toxicities (177–179).

## 4 Perspectives

**Figure 3:**
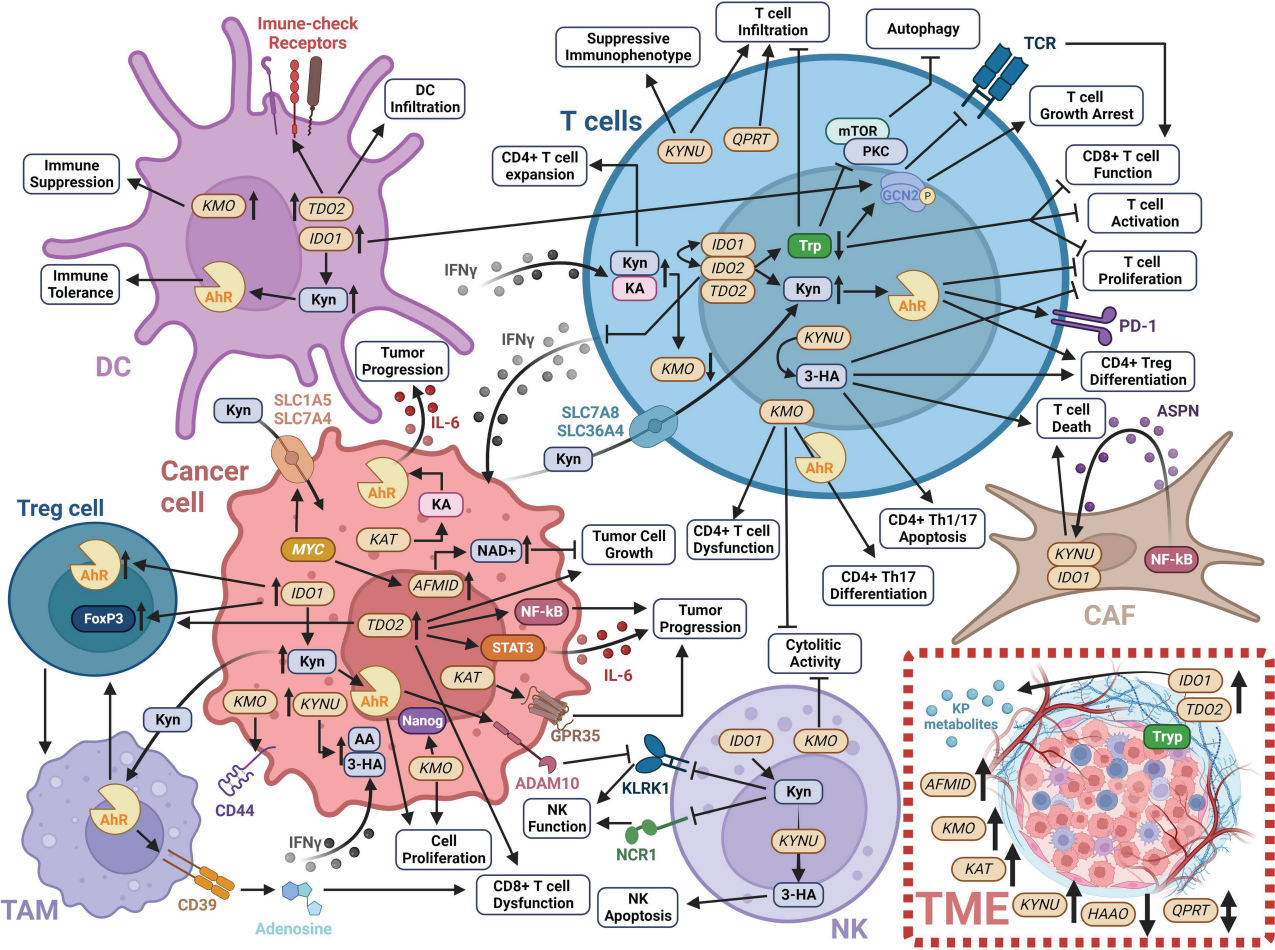
Schematic of the impact of KP enzymes and derived metabolites on modulating the tumor immune microenvironment. Detailed information is provided in Section 1 of this review. Created with BioRender.com. 3-HA, 3-hydroxyanthranilic acid; AA, anthranilic acid; ADAM10, ADAM metallopeptidase domain 10; Ahr, Aryl hydrocarbon receptor; AFMID, arylformamidase; ASPN, asporin; CAF, cancer-associated fibroblast; DC, dendritic cell; FoxP3, forkhead box P3; GCN2, general control non-derepressible 2; GPR35, G-protein-coupled receptor 35; HAAO, 3-hydroxyanthranilate 3;4-dioxygenase; IDO1, indoleamine 2;3-dioxygenase 1; IDO2, indoleamine 2;3-dioxygenase 2; IFNγ, interferon-gamma; IL-6, interleukin 6; KA, kynurenic acid; KAT, kynurenine aminotransferase; KLKR1, killer cell lectin like receptor K1; KMO, kynurenine 3-monooxygenase; KP, kynurenine pathway; KYNU, kynureninase; mTOR, mammalian target of rapamycin; NAD+, nicotinamide adenine dinucleotide; NCR1, natural cytotoxicity triggering receptor 1; NF-κB, nuclear factor kappa beta; NK, natural killer cell; PD-1, programmed cell death protein 1; PKC, protein kinase C; SLC1A5, solute carrier family 1 member 5; SLC36A4, solute carrier family 36 member 4; SLC7A5, solute carrier family 7 member 5; SLC7A8, solute carrier family 7 member 8; STAT3, signal transducer and activator of transcription 3; TAM, tumor-associated macrophages; TDO2, tryptophan 2;3-dioxygenase; TME, tumor microenvironment; Tryp, tryptophan; QPTR, quinolinic acid phosphoribosyl transferase.

Over the past few decades, there has been considerable advancement in our understanding of the cancer cell intrinsic and complex extrinsic functions of the KP in tumor development and progression (**Figure 3**). This progress has led to the development of several small molecule inhibitors that exploit multiple facets of the KP to achieve anti-tumor effects and the reversal of tumor-derived immune suppression (**Figure 3**; **Table 1**).

Despite these advances, clinical benefit resulting from targeting the KP pathway has been less than anticipated. Limited clinical success is likely to be multifactorial. For instance, advanced stage tumors have already undergone immunoediting to enable immune escape. Benefit of IDO inhibitors, or other KP inhibitors, may be most efficacious during early stages of disease when the immune system iteratively selects and/or promotes the generation of tumor cell variants with increasing capacities to survive immune attack, also referred to as the equilibrium phase (180).

Lack of clinical benefit may also be attributed to patient selection. Tumors that co-express IDO2 and TDO2 may be less responsive to selective IDO1 inhibitors. Consequently, molecular profiling of tumors with specific evaluation of IDO1, IDO2, and TDO2 expression may better select for patients that would benefit from selective IDO1 inhibitors or from broader pan-IDO/TDO2 inhibitors (**Table 1**). Recent findings implicating a nonenzymatic role of IDO1 in signaling and immune suppression (44) necessitates that existing or emerging inhibitors should also be evaluated for their effects on catalytic activity in addition to signaling functions to maximize potential for clinical efficacy.

It is also important to consider additional factors that may influence KP activity such as diet (67) and environmental contributors (181, 182). Liang and colleagues demonstrated that nicotine-derived nitrosaminoketone (NNK) upregulates IDO1 via α7 nicotinic acetylcholine receptor (α7nAChR)-mediated activation of the factor c-Jun in lung tumors. In A/J mice, NNK reduced CD8+ T cells and increased Tregs. Moreover, individuals with non-small cell lung cancer who smoked had higher tumoral IDO1 levels and lower Trp/Kyn ratios compared patients who never smoked, further suggesting that smoking impacts KP activity (181). Exposure to other environmental toxicants can also activate AhR signaling (182), which may upregulate IDO expression resulting in immune suppression (48).

The relevance of other downstream KP enzymes also warrants considerations. This is exemplified by our own findings that tumoral KYNU, but not IDO1, is selectively and frequently upregulated in NRF2 activated tumors and that elevated tumoral KYNU is associated with high immune cell infiltration across several cancer types (22, 23). One may infer that elevated tumoral KYNU promotes tumor immune cell infiltration by reducing levels of Kyn and increasing levels of immune inert anthranilic acid in the TME, as evidenced by studies using PEGylated-KYNU (8). Yet, elevated tumoral KYNU coincides with NRF2 activation, which can promote immune suppression by upregulating PD-L1 (183, 184). Crosstalk between cancer cells and TAMs in the TME can also activate NRF2 in TAMs to reshape the tumor immune microenvironment via multiple mechanisms including suppression of pro-inflammatory cytokines, increasing expression of PD-L1, macrophage colony-stimulating factor (M-CSF) and KYNU, and accelerating catabolism of cytotoxic-labile heme (185). Upregulation of tumoral KYNU, coupled with NRF2 activation may thus synergize to yield a TME that is highly immune suppressed. Whether therapeutic targeting of KYNU alone or in combination with ICI in NRF2-activated KYNU-high tumors reverses tumor immune suppression remains an area of ongoing inquiry.

In summary, the KP remains an active area of investigation, and insights gained from this study are expected reveal additional promising points actionable metabolic vulnerability within the pathway that can be targeted for anti-cancer treatment.

## Author contributions

RL-L: Visualization, Writing – original draft, Conceptualization. RD: Conceptualization, Writing – original draft. JV: Writing – review & editing. AS: Writing – review & editing. EO: Writing – review & editing. SH: Writing – review & editing. JF: Conceptualization, Investigation, Resources, Supervision, Writing – original draft.

## References

[1] Munn DH Mellor AL. Indoleamine 2,3 dioxygenase and metabolic control of immune responses. Trends Immunol (2013) 34(3):137–43. doi: 10.1016/j.it.2012.10.001 PMC3594632 23103127

[2] Solvay M Holfelder P Klaessens S Pilotte L Stroobant V Lamy J. Tryptophan depletion sensitizes the AHR pathway by increasing AHR expression and GCN2/LAT1-mediated kynurenine uptake, and potentiates induction of regulatory T lymphocytes. J Immunother Cancer (2023) 11(6):e006728. doi: 10.1136/jitc-2023-006728 37344101 PMC10314700

[3] Minhas PS Liu L Moon PK Joshi AU Dove C Mhatre S. Macrophage *de novo* NAD(+) synthesis specifies immune function in aging and inflammation. Nat Immunol (2019) 20(1):50–63. doi: 10.1038/s41590-018-0255-3 30478397 PMC6768398

[4] Badawy AA. Kynurenine pathway of tryptophan metabolism: regulatory and functional aspects. Int J Tryptophan Res (2017) 10. doi: 10.1177/1178646917691938 PMC5398323 28469468

[5] Badawy AA. Tryptophan metabolism and disposition in cancer biology and immunotherapy. Biosci Rep (2022) 42(11):1–27. doi: 10.1042/BSR20221682 PMC9653095 36286592

[6] Munn DH Sharma MD Baban B Harding HP Zhang Y Ron D. GCN2 kinase in T cells mediates proliferative arrest and anergy induction in response to indoleamine 2,3-dioxygenase. Immunity (2005) 22(5):633–42. doi: 10.1016/j.immuni.2005.03.013 15894280

[7] Cheong JE Sun L. Targeting the IDO1/TDO2-KYN-ahR pathway for cancer immunotherapy - challenges and opportunities. Trends Pharmacol Sci (2018) 39(3):307–25. doi: 10.1016/j.tips.2017.11.007 29254698

[8] Triplett TA Garrison KC Marshall N Donkor M Blazeck J Lamb C. Reversal of indoleamine 2,3-dioxygenase-mediated cancer immune suppression by systemic kynurenine depletion with a therapeutic enzyme. Nat Biotechnol (2018) 36(8):758–64. doi: 10.1038/nbt.4180 PMC6078800 30010674

[9] Platten M Wick W Van den Eynde BJ. Tryptophan catabolism in cancer: beyond IDO and tryptophan depletion. Cancer Res (2012) 72(21):5435–40. doi: 10.1158/0008-5472.CAN-12-0569 23090118

[10] Long GV Dummer R Hamid O Gajewski TF Caglevic C Dalle S. Epacadostat plus pembrolizumab versus placebo plus pembrolizumab in patients with unresectable or metastatic melanoma (ECHO-301/KEYNOTE-252): a phase 3, randomised, double-blind study. Lancet Oncol (2019) 20(8):1083–97. doi: 10.1016/S1470-2045(19)30274-8 31221619

[11] Beatty GL O'Dwyer PJ Clark J Shi JG Bowman KJ Scherle PA. First-in-human phase I study of the oral inhibitor of indoleamine 2,3-dioxygenase-1 epacadostat (INCB024360) in patients with advanced solid Malignancies. Clin Cancer Res (2017) 23(13):3269–76. doi: 10.1158/1078-0432.CCR-16-2272 PMC5496788 28053021

[12] Yue EW Sparks R Polam P Modi D Douty B Wayland B. INCB24360 (Epacadostat), a highly potent and selective indoleamine-2,3-dioxygenase 1 (IDO1) inhibitor for immuno-oncology. ACS Med Chem Lett (2017) 8(5):486–91. doi: 10.1021/acsmedchemlett.6b00391 PMC5430407 28523098

[13] Mitchell TC Hamid O Smith DC Bauer TM Wasser JS Olszanski AJ. Epacadostat plus pembrolizumab in patients with advanced solid tumors: phase I results from a multicenter, open-label phase I/II trial (ECHO-202/KEYNOTE-037). J Clin Oncol (2018) 36(32):3223–30. doi: 10.1200/JCO.2018.78.9602 PMC6225502 30265610

[14] Gibney GT Hamid O Lutzky J Olszanski AJ Mitchell TC Gajewski TF. Phase 1/2 study of epacadostat in combination with ipilimumab in patients with unresectable or metastatic melanoma. J Immunother Cancer (2019) 7(1):80. doi: 10.1186/s40425-019-0562-8 30894212 PMC6425606

[15] Lara P Bauer T Hamid O Smith D Gajewski T Gangadhar T. Epacadostat plus pembrolizumab in patients with advanced RCC: Preliminary phase I/II results from ECHO-202/KEYNOTE-037. J Clin Oncol (2017) 35:4515. doi: 10.1200/JCO.2017.35.15_suppl.4515

[16] Smith D Gajewski T Hamid O Wasser J Olszanski A Patel S. Epacadostat plus pembrolizumab in patients with advanced urothelial carcinoma: Preliminary phase I/II results of ECHO-202/KEYNOTE-037. J Clin Oncol (2017) 35:4503. doi: 10.1200/JCO.2017.35.15_suppl.4503

[17] Prendergast GC Malachowski WP DuHadaway JB Muller AJ. Discovery of IDO1 inhibitors: from bench to bedside. Cancer Res (2017) 77(24):6795–811. doi: 10.1016/bs.ircmb.2017.07.004 PMC6021761 29247038

[18] Metz R Rust S Duhadaway JB Mautino MR Munn DH Vahanian NN. IDO inhibits a tryptophan sufficiency signal that stimulates mTOR: A novel IDO effector pathway targeted by D-1-methyl-tryptophan. Oncoimmunology (2012) 1(9):1460–8. doi: 10.4161/onci.21716 PMC3525601 23264892

[19] Jung KH LoRusso P Burris H Gordon M Bang YJ Hellmann MD. Phase I study of the indoleamine 2,3-dioxygenase 1 (IDO1) inhibitor navoximod (GDC-0919) administered with PD-L1 inhibitor (Atezolizumab) in advanced solid tumors. Clin Cancer Res (2019) 25(11):3220–8. doi: 10.1158/1078-0432.CCR-18-2740 PMC7980952 30770348

[20] Ebata T Shimizu T Fujiwara Y Tamura K Kondo S Iwasa S. Phase I study of the indoleamine 2,3-dioxygenase 1 inhibitor navoximod (GDC-0919) as monotherapy and in combination with the PD-L1 inhibitor atezolizumab in Japanese patients with advanced solid tumours. Invest New Drugs (2020) 38(2):468–77. doi: 10.1007/s10637-019-00787-3 PMC7066107 31124055

[21] Burris H Gordon M Hellmann M Lorusso P Emens L Hodi F. A phase Ib dose escalation study of combined inhibition of IDO1 (GDC-0919) and PD-L1 (atezolizumab) in patients (pts) with locally advanced or metastatic solid tumors. J Clin Oncol (2017) 35:105. doi: 10.1200/JCO.2017.35.15_suppl.105

[22] Fahrmann JF Tanaka I Irajizad E Mao X Dennison JB Murage E. Mutational activation of the NRF2 pathway upregulates kynureninase resulting in tumor immunosuppression and poor outcome in lung adenocarcinoma. Cancers (Basel) (2022) 14(10):1–29. doi: 10.3390/cancers14102543 PMC9139317 35626147

[23] Leon-Letelier RA Abdel Sater AH Chen Y Park S Wu R Irajizad E. Kynureninase upregulation is a prominent feature of NFR2-activated cancers and is associated with tumor immunosuppression and poor prognosis. Cancers (Basel) (2023) 15(3):1–14. doi: 10.3390/cancers15030834 PMC9913753 36765792

[24] Yan Y Zhang GX Gran B Fallarino F Yu S Li H. IDO upregulates regulatory T cells *via* tryptophan catabolite and suppresses encephalitogenic T cell responses in experimental autoimmune encephalomyelitis. J Immunol (2010) 185(10):5953–61. doi: 10.4049/jimmunol.1001628 PMC2998795 20944000

[25] Rad Pour S Morikawa H Kiani NA Yang M Azimi A Shafi G. Exhaustion of CD4+ T-cells mediated by the kynurenine pathway in melanoma. Sci Rep (2019) 9(1):12150. doi: 10.1038/s41598-019-48635-x 31434983 PMC6704156

[26] Ray A Song Y Du T Tai YT Chauhan D Anderson KC. Targeting tryptophan catabolic kynurenine pathway enhances antitumor immunity and cytotoxicity in multiple myeloma. Leukemia (2020) 34(2):567–77. doi: 10.1038/s41375-019-0558-x PMC7132142 31462737

[27] Orecchini E Belladonna ML Pallotta MT Volpi C Zizi L Panfili E. The signaling function of IDO1 incites the Malignant progression of mouse B16 melanoma. Oncoimmunology (2023) 12(1):2170095. doi: 10.1080/2162402X.2023.2170095 36733497 PMC9888476

[28] Zhai L Ladomersky E Lenzen A Nguyen B Patel R Lauing KL. IDO1 in cancer: a Gemini of immune checkpoints. Cell Mol Immunol (2018) 15(5):447–57. doi: 10.1038/cmi.2017.143 PMC6068130 29375124

[29] Rohatgi N Ghoshdastider U Baruah P Kulshrestha T Skanderup AJ. A pan-cancer metabolic atlas of the tumor microenvironment. Cell Rep (2022) 39(6):110800. doi: 10.1016/j.celrep.2022.110800 35545044

[30] Yu CP Fu SF Chen X Ye J Ye Y Kong LD. The clinicopathological and prognostic significance of IDO1 expression in human solid tumors: evidence from a systematic review and meta-analysis. Cell Physiol Biochem (2018) 49(1):134–43. doi: 10.1159/000492849 30134237

[31] Brandacher G Perathoner A Ladurner R Schneeberger S Obrist P Winkler C. Prognostic value of indoleamine 2,3-dioxygenase expression in colorectal cancer: effect on tumor-infiltrating T cells. Clin Cancer Res (2006) 12(4):1144–51. doi: 10.1158/1078-0432.CCR-05-1966 16489067

[32] Fallarino F Grohmann U You S McGrath BC Cavener DR Vacca C. The combined effects of tryptophan starvation and tryptophan catabolites down-regulate T cell receptor zeta-chain and induce a regulatory phenotype in naive T cells. J Immunol (2006) 176(11):6752–61. doi: 10.4049/jimmunol.176.11.6752 16709834

[33] Liu Y Liang X Dong W Fang Y Lv J Zhang T. Tumor-repopulating cells induce PD-1 expression in CD8+ T cells by transferring kynurenine and ahR activation. Cancer Cell (2018) 33(3):480–94.e7. doi: 10.1016/j.ccell.2018.02.005 29533786

[34] Qin R Zhao C Wang CJ Xu W Zhao JY Lin Y. Tryptophan potentiates CD8(+) T cells against cancer cells by TRIP12 tryptophanylation and surface PD-1 downregulation. J Immunother Cancer (2021) 9(7):e002840. doi: 10.1136/jitc-2021-002840 34326168 PMC8323461

[35] Corradi G Bassani B Simonetti G Sangaletti S Vadakekolathu J Fontana MC. Release of IFNgamma by acute myeloid leukemia cells remodels bone marrow immune microenvironment by inducing regulatory T cells. Clin Cancer Res (2022) 28(14):3141–55. doi: 10.1158/1078-0432.CCR-21-3594 35349670

[36] Park A Yang Y Lee Y Kim MS Park YJ Jung H. Indoleamine-2,3-dioxygenase in thyroid cancer cells suppresses natural killer cell function by inhibiting NKG2D and NKp46 expression *via* STAT signaling pathways. J Clin Med (2019) 8(6):842. doi: 10.3390/jcm8060842 31212870 PMC6617210

[37] Chiesa MD Carlomagno S Frumento G Balsamo M Cantoni C Conte R. The tryptophan catabolite l-kynurenine inhibits the surface expression of NKp46- and NKG2D-activating receptors and regulates NK-cell function. Blood (2006) 108(13):4118–25. doi: 10.1182/blood-2006-03-006700 16902152

[38] Fang X Guo L Xing Z Shi L Liang H Li A. IDO1 can impair NK cells function against non-small cell lung cancer by downregulation of NKG2D Ligand *via* ADAM10. Pharmacol Res (2022) 177:106132. doi: 10.1016/j.phrs.2022.106132 35183714

[39] Takenaka MC Gabriely G Rothhammer V Mascanfroni ID Wheeler MA Chao CC. Control of tumor-associated macrophages and T cells in glioblastoma *via* AHR and CD39. Nat Neurosci (2019) 22(5):729–40. doi: 10.1038/s41593-019-0370-y PMC8052632 30962630

[40] Campesato LF Budhu S Tchaicha J Weng C-H Gigoux M Cohen IJ. Blockade of the AHR restricts a Treg-macrophage suppressive axis induced by L-Kynurenine. Nat Commun (2020) 11(1):4011. doi: 10.1038/s41467-020-17750-z 32782249 PMC7419300

[41] Pallotta MT Orabona C Volpi C Vacca C Belladonna ML Bianchi R. Indoleamine 2,3-dioxygenase is a signaling protein in long-term tolerance by dendritic cells. Nat Immunol (2011) 12(9):870–8. doi: 10.1038/ni.2077 21804557

[42] Pallotta MT Rossini S Suvieri C Coletti A Orabona C Macchiarulo A. Indoleamine 2,3-dioxygenase 1 (IDO1): an up-to-date overview of an eclectic immunoregulatory enzyme. FEBS J (2022) 289(20):6099–118. doi: 10.1111/febs.16086 PMC9786828 34145969

[43] Iacono A Pompa A De Marchis F Panfili E Greco FA Coletti A. Class IA PI3Ks regulate subcellular and functional dynamics of IDO1. EMBO Rep (2020) 21(12):e49756. doi: 10.15252/embr.201949756 33159421 PMC7726814

[44] Zhai L Bell A Ladomersky E Lauing KL Bollu L Nguyen B. Tumor cell IDO enhances immune suppression and decreases survival indepndent of tryptophan metabolism in glioblastoma. Clin Cancer Res (2021) 27(23):6514–28. doi: 10.1158/1078-0432.CCR-21-1392 PMC8639612 34479957

[45] Metz R Duhadaway JB Kamasani U Laury-Kleintop L Muller AJ Prendergast GC. Novel tryptophan catabolic enzyme IDO2 is the preferred biochemical target of the antitumor indoleamine 2,3-dioxygenase inhibitory compound D-1-methyl-tryptophan. Cancer Res (2007) 67(15):7082–7. doi: 10.1158/0008-5472.CAN-07-1872 17671174

[46] Ball HJ Sanchez-Perez A Weiser S Austin CJ Astelbauer F Miu J. Characterization of an indoleamine 2,3-dioxygenase-like protein found in humans and mice. Gene (2007) 396(1):203–13. doi: 10.1016/j.gene.2007.04.010 17499941

[47] Kado SY Bein K Castaneda AR Pouraryan AA Garrity N Ishihara Y. Regulation of IDO2 by the aryl hydrocarbon receptor (AhR) in breast cancer. Cells (2023) 12(10):1433. doi: 10.3390/cells12101433 37408267 PMC10216785

[48] Vogel CF Goth SR Dong B Pessah IN Matsumura F. Aryl hydrocarbon receptor signaling mediates expression of indoleamine 2,3-dioxygenase. Biochem Biophys Res Commun (2008) 375(3):331–5. doi: 10.1016/j.bbrc.2008.07.156 PMC2583959 18694728

[49] Li Q Harden JL Anderson CD Egilmez NK. Tolerogenic phenotype of IFN-γ-induced IDO+ Dendritic cells is maintained *via* an autocrine IDO-kynurenine/ahR-IDO loop. J Immunol (2016) 197(3):962–70. doi: 10.4049/jimmunol.1502615 27316681

[50] Liu Y Zhang Y Zheng X Zhang X Wang H Li Q. Gene silencing of indoleamine 2,3-dioxygenase 2 in melanoma cells induces apoptosis through the suppression of NAD+ and inhibits in *vivo* tumor growth. Oncotarget (2016) 7(22):32329–40. doi: 10.18632/oncotarget.8617 PMC5078016 27058624

[51] Mo B Zhao X Wang Y Jiang X Liu D Cai H. Pan-cancer analysis, providing a reliable basis for IDO2 as a prognostic biomarker and target for immunotherapy. Oncologie (2023) 25(1):17–35. doi: 10.1515/oncologie-2022-1026

[52] Yamasuge W Yamamoto Y Fujigaki H Hoshi M Nakamoto K Kunisawa K. Indoleamine 2,3-dioxygenase 2 depletion suppresses tumor growth in a mouse model of Lewis lung carcinoma. Cancer Sci (2019) 110(10):3061–7. doi: 10.1111/cas.14179 PMC6778659 31444833

[53] Abd El-Fattah EE. IDO/kynurenine pathway in cancer: possible therapeutic approaches. J Transl Med (2022) 20(1):347. doi: 10.1186/s12967-022-03554-w 35918736 PMC9344609

[54] Yuasa HJ Ball HJ Ho YF Austin CJD Whittington CM Belov K. Characterization and evolution of vertebrate indoleamine 2, 3-dioxygenases: IDOs from monotremes and marsupials. Comp Biochem Physiol Part B: Biochem Mol Biol (2009) 153(2):137–44. doi: 10.1016/j.cbpb.2009.02.002 19402226

[55] Metz R Smith C DuHadaway JB Chandler P Baban B Merlo LM. IDO2 is critical for IDO1-mediated T-cell regulation and exerts a non-redundant function in inflammation. Int Immunol (2014) 26(7):357–67. doi: 10.1093/intimm/dxt073 PMC4432394 24402311

[56] Merlo LMF DuHadaway JB Montgomery JD Peng WD Murray PJ Prendergast GC. Differential roles of IDO1 and IDO2 in T and B cell inflammatory immune responses. Front Immunol (2020) 11:1861. doi: 10.3389/fimmu.2020.01861 32973768 PMC7461966

[57] Merlo LMF Peng W DuHadaway JB Montgomery JD Prendergast GC Muller AJ. The immunomodulatory enzyme IDO2 mediates autoimmune arthritis through a nonenzymatic mechanism. J Immunol (2022) 208(3):571–81. doi: 10.4049/jimmunol.2100705 PMC8770583 34965962

[58] Cui J Tian Y Liu T Lin X Li L Li Z. Pancancer analysis of revealed TDO2 as a biomarker of prognosis and immunotherapy. Dis Markers (2022) 2022:5447017. doi: 10.1155/2022/5447017 36118672 PMC9481368

[59] Liu H Xiang Y Zong QB Dai ZT Wu H Zhang HM. TDO2 modulates liver cancer cell migration and invasion via the Wnt5a pathway. Int J Oncol (2022) 60(6):72. doi: 10.3892/ijo.2022.5362 35475491

[60] Zhao L Wang B Yang C Lin Y Zhang Z Wang S. TDO2 knockdown inhibits colorectal cancer progression *via* TDO2-KYNU-AhR pathway. Gene (2021) 792:145736. doi: 10.1016/j.gene.2021.145736 34051337

[61] Liu Q Zhai J Kong X Wang X Wang Z Fang Y. Comprehensive analysis of the expression and prognosis for TDO2 in breast cancer. Mol Ther Oncolytics (2020) 17:153–68. doi: 10.1016/j.omto.2020.03.013 PMC7178007 32346606

[62] Kudo T Prentzell MT Mohapatra SR Sahm F Zhao Z Grummt I. Constitutive expression of the immunosuppressive tryptophan dioxygenase TDO2 in glioblastoma is driven by the transcription factor C/EBPβ. Front Immunol (2020) 11:657. doi: 10.3389/fimmu.2020.00657 32477324 PMC7239998

[63] Miyazaki T Chung S Sakai H Ohata H Obata Y Shiokawa D. Stemness and immune evasion conferred by the TDO2-AHR pathway are associated with liver metastasis of colon cancer. Cancer Sci (2022) 113(1):170–81. doi: 10.1111/cas.15182 PMC8748246 34714577

[64] Gutiérrez-Vázquez C Quintana FJ. Regulation of the immune response by the aryl hydrocarbon receptor. Immunity (2018) 48(1):19–33. doi: 10.1016/j.immuni.2017.12.012 29343438 PMC5777317

[65] Hu S Lu H Xie W Wang D Shan Z Xing X. TDO2+ myofibroblasts mediate immune suppression in Malignant transformation of squamous cell carcinoma. J Clin Invest (2022) 132(19):e157649. doi: 10.1172/JCI157649 35972800 PMC9525123

[66] Kanai M Funakoshi H Takahashi H Hayakawa T Mizuno S Matsumoto K. Tryptophan 2,3-dioxygenase is a key modulator of physiological neurogenesis and anxiety-related behavior in mice. Mol Brain (2009) 2:8. doi: 10.1186/1756-6606-2-8 19323847 PMC2673217

[67] Schramme F Crosignani S Frederix K Hoffmann D Pilotte L Stroobant V. Inhibition of tryptophan-dioxygenase activity increases the antitumor efficacy of immune checkpoint inhibitors. Cancer Immunol Res (2020) 8(1):32–45. doi: 10.1158/2326-6066.CIR-19-0041 31806638

[68] Wu Z Yan L Lin J Ke K Yang W. Constitutive TDO2 expression promotes liver cancer progression by an autocrine IL-6 signaling pathway. Cancer Cell Int (2021) 21(1):538. doi: 10.1186/s12935-021-02228-9 34657635 PMC8522106

[69] Lee R Li J Li J Wu CJ Jiang S Hsu WH. Synthetic essentiality of tryptophan 2,3-dioxygenase 2 in APC-mutated colorectal cancer. Cancer Discovery (2022) 12(7):1702–17. doi: 10.1158/2159-8290.CD-21-0680 PMC9262860 35537038

[70] Perez-Castro L Garcia R Venkateswaran N Barnes S Conacci-Sorrell M. Tryptophan and its metabolites in normal physiology and cancer etiology. FEBS J (2023) 290(1):7–27. doi: 10.1111/febs.16245 34687129 PMC9883803

[71] Venkateswaran N Conacci-Sorrell M. Kynurenine: an oncometabolite in colon cancer. Cell Stress (2020) 4(1):24–6. doi: 10.15698/cst2020.01.210 PMC6946015 31922097

[72] Venkateswaran N Lafita-Navarro MC Hao YH Kilgore JA Perez-Castro L Braverman J. MYC promotes tryptophan uptake and metabolism by the kynurenine pathway in colon cancer. Genes Dev (2019) 33(17-18):1236–51. doi: 10.1101/gad.327056.119 PMC6719621 31416966

[73] Lin KT Ma WK Scharner J Liu YR Krainer AR. A human-specific switch of alternatively spliced AFMID isoforms contributes to TP53 mutations and tumor recurrence in hepatocellular carcinoma. Genome Res (2018) 28(3):275–84. doi: 10.1101/gr.227181.117 PMC5848607 29449409

[74] Tummala KS Gomes AL Yilmaz M Graña O Bakiri L Ruppen I. Inhibition of *de novo* NAD(+) synthesis by oncogenic URI causes liver tumorigenesis through DNA damage. Cancer Cell (2014) 26(6):826–39. doi: 10.1016/j.ccell.2014.10.002 25453901

[75] Tripathi SC Fahrmann JF Vykoukal JV Dennison JB Hanash SM. Targeting metabolic vulnerabilities of cancer: Small molecule inhibitors in clinic. Cancer Rep (Hoboken) (2019) 2(1):e1131. doi: 10.1073/pnas.1521812113 32721114 PMC7941539

[76] Wang M Wang Y Zhang M Duan Q Chen C Sun Q. Kynureninase contributes to the pathogenesis of psoriasis through pro-inflammatory effect. J Cell Physiol (2022) 237(1):1044–56. doi: 10.1002/jcp.30587 34553380

[77] Mohapatra SR Sadik A Tykocinski LO Dietze J Poschet G Heiland I. Hypoxia inducible factor 1alpha inhibits the expression of immunosuppressive tryptophan-2,3-dioxygenase in glioblastoma. Front Immunol (2019) 10:2762. doi: 10.3389/fimmu.2019.02762 31866995 PMC6905408

[78] Li Y Wang M Zhao L Liang C Li W. KYNU-related transcriptome profile and clinical outcome from 2994 breast tumors. Heliyon (2023) 9(6):e17216. doi: 10.1016/j.heliyon.2023.e17216 37383199 PMC10293725

[79] Itoh G Takagane K Fukushi Y Kuriyama S Umakoshi M Goto A. Cancer-associated fibroblasts educate normal fibroblasts to facilitate cancer cell spreading and T-cell suppression. Mol Oncol (2022) 16(1):166–87. doi: 10.1002/1878-0261.13077 PMC8732346 34379869

[80] Kesarwani P Kant S Zhao Y Prabhu A Buelow KL Miller CR. Quinolinate promotes macrophage-induced immune tolerance in glioblastoma through the NMDAR/PPARgamma signaling axis. Nat Commun (2023) 14(1):1459. doi: 10.1038/s41467-023-37170-z 36927729 PMC10020159

[81] Heng B Bilgin AA Lovejoy DB Tan VX Milioli HH Gluch L. Differential kynurenine pathway metabolism in highly metastatic aggressive breast cancer subtypes: beyond IDO1-induced immunosuppression. Breast Cancer Res (2020) 22(1):113. doi: 10.1186/s13058-020-01351-1 33109232 PMC7590459

[82] Liu D Liang CH Huang B Zhuang X Cui W Yang L. Tryptophan metabolism acts as a new anti-ferroptotic pathway to mediate tumor growth. Adv Sci (Weinh) (2023) 10(6):e2204006. doi: 10.1002/advs.202204006 36627132 PMC9951368

[83] Weber WP Feder-Mengus C Chiarugi A Rosenthal R Reschner A Schumacher R. Differential effects of the tryptophan metabolite 3-hydroxyanthranilic acid on the proliferation of human CD8+ T cells induced by TCR triggering or homeostatic cytokines. Eur J Immunol (2006) 36(2):296–304. doi: 10.1002/eji.200535616 16385630

[84] Amaral M Levy C Heyes DJ Lafite P Outeiro TF Giorgini F. Structural basis of kynurenine 3-monooxygenase inhibition. Nature (2013) 496(7445):382–5. doi: 10.1038/nature12039 PMC3736096 23575632

[85] Huang TT Tseng LM Chen JL Chu PY Lee CH Huang CT. Kynurenine 3-monooxygenase upregulates pluripotent genes through beta-catenin and promotes triple-negative breast cancer progression. EBioMedicine (2020) 54:102717. doi: 10.1016/j.ebiom.2020.102717 32268268 PMC7191260

[86] Liu CY Huang TT Chen JL Chu PY Lee CH Lee HC. Significance of kynurenine 3-monooxygenase expression in colorectal cancer. Front Oncol (2021) 11:620361. doi: 10.3389/fonc.2021.620361 33937026 PMC8085544

[87] Shi Z Gan G Gao X Chen F Mi J. Kynurenine catabolic enzyme KMO regulates HCC growth. Clin Transl Med (2022) 12(2):e697. doi: 10.1002/ctm2.697 35184386 PMC8858614

[88] Vazquez Cervantes GI Pineda B Ramirez Ortega D Salazar A Gonzalez Esquivel DF Rembao D. Kynurenine monooxygenase expression and activity in human astrocytomas. Cells (2021) 10(8):2028. doi: 10.3390/cells10082028 34440798 PMC8393384

[89] Tsang YW Liao CH Ke CH Tu CW Lin CS. Integrated molecular characterization to reveal the association between kynurenine 3-monooxygenase expression and tumorigenesis in human breast cancers. J Pers Med (2021) 11(10):948. doi: 10.3390/jpm11100948 34683090 PMC8539700

[90] Lai MH Liao CH Tsai NM Chang KF Liu CC Chiu YH. Surface expression of kynurenine 3-monooxygenase promotes proliferation and metastasis in triple-negative breast cancers. Cancer Control (2021) 28. doi: 10.1177/10732748211009245 PMC8204454 33887987

[91] Yu P Li Z Zhang L Tagle DA Cai T. Characterization of kynurenine aminotransferase III, a novel member of a phylogenetically conserved KAT family. Gene (2006) 365:111–8. doi: 10.1016/j.gene.2005.09.034 16376499

[92] Walczak K Wnorowski A Turski WA Plech T. Kynurenic acid and cancer: facts and controversies. Cell Mol Life Sci (2020) 77(8):1531–50. doi: 10.1007/s00018-019-03332-w PMC7162828 31659416

[93] Goldsmith ZG Dhanasekaran DN. G protein regulation of MAPK networks. Oncogene (2007) 26(22):3122–42. doi: 10.1038/sj.onc.1210407 17496911

[94] Walczak K Turski WA Rajtar G. Kynurenic acid inhibits colon cancer proliferation in *vitro*: effects on signaling pathways. Amino Acids (2014) 46(10):2393–401. doi: 10.1007/s00726-014-1790-3 PMC4168223 25012123

[95] Walczak K Turski WA Rzeski W. Kynurenic acid enhances expression of p21 Waf1/Cip1 in colon cancer HT-29 cells. Pharmacol Rep (2012) 64(3):745–50. doi: 10.1016/S1734-1140(12)70870-8 22814028

[96] Walczak K Zurawska M Kiś J Starownik R Zgrajka W Bar K. Kynurenic acid in human renal cell carcinoma: its antiproliferative and antimigrative action on Caki-2 cells. Amino Acids (2012) 43(4):1663–70. doi: 10.1007/s00726-012-1247-5 22349835

[97] Moroni F Cozzi A Sili M Mannaioni G. Kynurenic acid: a metabolite with multiple actions and multiple targets in brain and periphery. J Neural Transm (2012) 119(2):133–9. doi: 10.1007/s00702-011-0763-x 22215208

[98] Pagano E Elias JE Schneditz G Saveljeva S Holland LM Borrelli F. Activation of the GPR35 pathway drives angiogenesis in the tumour microenvironment. Gut (2022) 71(3):509–20. doi: 10.1136/gutjnl-2020-323363 PMC8862021 33758004

[99] DiNatale BC Murray IA Schroeder JC Flaveny CA Lahoti TS Laurenzana EM. Kynurenic acid is a potent endogenous aryl hydrocarbon receptor ligand that synergistically induces interleukin-6 in the presence of inflammatory signaling. Toxicol Sci (2010) 115(1):89–97. doi: 10.1093/toxsci/kfq024 20106948 PMC2855350

[100] Fisher DT Appenheimer MM Evans SS. The two faces of IL-6 in the tumor microenvironment. Semin Immunol (2014) 26(1):38–47. doi: 10.1016/j.smim.2014.01.008 24602448 PMC3970580

[101] Sadik A Somarribas Patterson LF Ozturk S Mohapatra SR Panitz V Secker PF. IL4I1 is a metabolic immune checkpoint that activates the AHR and promotes tumor progression. Cell (2020) 182(5):1252–70.e34. doi: 10.1016/j.cell.2020.07.038 32818467

[102] Huang YW Luo J Weng YI Mutch DG Goodfellow PJ Miller DS. Promoter hypermethylation of CIDEA, HAAO and RXFP3 associated with microsatellite instability in endometrial carcinomas. Gynecol Oncol (2010) 117(2):239–47. doi: 10.1016/j.ygyno.2010.02.006 PMC2849881 20211485

[103] Li Y Meng L Shi T Ren J Deng Q. Diagnosis and prognosis potential of four gene promoter hypermethylation in prostate cancer. Cell Biol Int (2021) 45(1):117–26. doi: 10.1002/cbin.11474 32991011

[104] Sahm F Oezen I Opitz CA Radlwimmer B von Deimling A Ahrendt T. The endogenous tryptophan metabolite and NAD+ precursor quinolinic acid confers resistance of gliomas to oxidative stress. Cancer Res (2013) 73(11):3225–34. doi: 10.1158/0008-5472.CAN-12-3831 23548271

[105] Liu CL Cheng SP Chen MJ Lin CH Chen SN Kuo YH. Quinolinate phosphoribosyltransferase promotes invasiveness of breast cancer through myosin light chain phosphorylation. Front Endocrinol (Lausanne) (2020) 11:621944. doi: 10.3389/fendo.2020.621944 33613454 PMC7890081

[106] Yue Z Shusheng J Hongtao S Shu Z Lan H Qingyuan Z. Silencing DSCAM-AS1 suppresses the growth and invasion of ER-positive breast cancer cells by downregulating both DCTPP1 and QPRT. Aging (Albany NY) (2020) 12(14):14754–74. doi: 10.18632/aging.103538 PMC7425442 32716908

[107] Ullmark T Montano G Jarvstrat L Jernmark Nilsson H Hakansson E Drott K. Anti-apoptotic quinolinate phosphoribosyltransferase (QPRT) is a target gene of Wilms' tumor gene 1 (WT1) protein in leukemic cells. Biochem Biophys Res Commun (2017) 482(4):802–7. doi: 10.1016/j.bbrc.2016.11.114 27889611

[108] Niu YC Tong J Shi XF Zhang T. MicroRNA-654-3p enhances cisplatin sensitivity by targeting QPRT and inhibiting the PI3K/AKT signaling pathway in ovarian cancer cells. Exp Ther Med (2020) 20(2):1467–79. doi: 10.3892/etm.2020.8878 PMC7388328 32742380

[109] Thongon N Zucal C D'Agostino VG Tebaldi T Ravera S Zamporlini F. Cancer cell metabolic plasticity allows resistance to NAMPT inhibition but invariably induces dependence on LDHA. Cancer Metab (2018) 6:1. doi: 10.1186/s40170-018-0174-7 29541451 PMC5844108

[110] Hornigold N Dunn KR Craven RA Zougman A Trainor S Shreeve R. Dysregulation at multiple points of the kynurenine pathway is a ubiquitous feature of renal cancer: implications for tumour immune evasion. Br J Cancer (2020) 123(1):137–47. doi: 10.1038/s41416-020-0874-y PMC7341846 32390008

[111] Hamid O Gajewski TF Frankel AE Bauer TM Olszanski AJ Luke JJ. 1214O - Epacadostat plus pembrolizumab in patients with advanced melanoma: Phase 1 and 2 efficacy and safety results from ECHO-202/KEYNOTE-037. Ann Oncol (2017) 28:v428–v9. doi: 10.1093/annonc/mdx377.001

[112] Gangadhar T Schneider B Bauer T Wasser J Spira A Patel S. Efficacy and safety of epacadostat plus pembrolizumab treatment of NSCLC: Preliminary phase I/II results of ECHO-202/KEYNOTE-037. J Clin Oncol (2017) 35:9014–. doi: 10.1200/JCO.2017.35.15_suppl.9014

[113] Powderly JD Klempner SJ Naing A Bendell J Garrido-Laguna I Catenacci DVT. Epacadostat plus pembrolizumab and chemotherapy for advanced solid tumors: results from the phase I/II ECHO-207/KEYNOTE-723 study. Oncologist (2022) 27(11):905–e848. doi: 10.1093/oncolo/oyac174 36156099 PMC9632315

[114] Kelly CM Qin LX Whiting KA Richards AL Avutu V Chan JE. A phase 2 study of epacadostat and pembrolizumab in patients with advanced sarcoma. Clin Cancer Res (2023) 29(11):2043–51. doi: 10.1158/1078-0432.c.6604903.v2 PMC10752758 36971773

[115] Perez R Riese M Lewis K Saleh M Daud A Berlin J. Epacadostat plus nivolumab in patients with advanced solid tumors: Preliminary phase I/II results of ECHO-204. J Clin Oncol (2017) 35:3003. doi: 10.1200/JCO.2017.35.15_suppl.3003

[116] Nayak-Kapoor A Hao Z Sadek R Dobbins R Marshall L Vahanian NN. Phase Ia study of the indoleamine 2,3-dioxygenase 1 (IDO1) inhibitor navoximod (GDC-0919) in patients with recurrent advanced solid tumors. J Immunother Cancer (2018) 6(1):61. doi: 10.1186/s40425-018-0351-9 29921320 PMC6009946

[117] Zakharia Y Johnson TS Colman H Vahanian NN Link CJ Kennedy E. A phase I/II study of the combination of indoximod and temozolomide for adult patients with temozolomide-refractory primary Malignant brain tumors. J Clin Oncol (2014) 32(15_suppl):TPS2107–TPS. doi: 10.1200/jco.2014.32.15_suppl.tps2107

[118] Soliman HH Minton SE Han HS Ismail-Khan R Neuger A Khambati F. A phase I study of indoximod in patients with advanced Malignancies. Oncotarget (2016) 7(16):22928–38. doi: 10.18632/oncotarget.8216 PMC5008412 27008709

[119] Soliman HH Jackson E Neuger T Dees EC Harvey RD Han H. A first in man phase I trial of the oral immunomodulator, indoximod, combined with docetaxel in patients with metastatic solid tumors. Oncotarget (2014) 5(18):8136–46. doi: 10.18632/oncotarget.2357 PMC4226672 25327557

[120] Jha GG Gupta S Tagawa ST Koopmeiners JS Vivek S Dudek AZ. A phase II randomized, double-blind study of sipuleucel-T followed by IDO pathway inhibitor, indoximod, or placebo in the treatment of patients with metastatic castration resistant prostate cancer (mCRPC). J Clin Oncol (2017) 35(15_suppl):3066. doi: 10.1200/JCO.2017.35.15_suppl.3066

[121] Zakharia Y McWilliams RR Rixe O Drabick J Shaheen MF Grossmann KF. Phase II trial of the IDO pathway inhibitor indoximod plus pembrolizumab for the treatment of patients with advanced melanoma. J Immunother Cancer (2021) 9(6):1–9. doi: 10.1136/jitc2020-002057 PMC8202104 34117113

[122] Cherney EC Zhang L Nara S Zhu X Gullo-Brown J Maley D. Discovery and preclinical evaluation of BMS-986242, a potent, selective inhibitor of indoleamine-2,3-dioxygenase 1. ACS Med Chem Lett (2021) 12(2):288–94. doi: 10.1021/acsmedchemlett.0c00668 PMC7883469 33603977

[123] Li P Wu R Li K Yuan W Zeng C Zhang Y. IDO inhibition facilitates antitumor immunity of Vγ9Vδ2 T cells in triple-negative breast cancer. Front Oncol (2021) 11:679517. doi: 10.3389/fonc.2021.679517 34381711 PMC8351331

[124] Pham KN Yeh SR. Mapping the binding trajectory of a suicide inhibitor in human indoleamine 2,3-dioxygenase 1. J Am Chem Soc (2018) 140(44):14538–41. doi: 10.1021/jacs.8b07994 PMC6430111 30347977

[125] Kim DK Synn CB Yang SM Kang S Baek S Oh SW. YH29407 with anti-PD-1 ameliorates anti-tumor effects *via* increased T cell functionality and antigen presenting machinery in the tumor microenvironment. Front Chem (2022) 10:998013. doi: 10.3389/fchem.2022.998013 36545214 PMC9761775

[126] Crosignani S Bingham P Bottemanne P Cannelle H Cauwenberghs S Cordonnier M. Discovery of a novel and selective indoleamine 2,3-dioxygenase (IDO-1) inhibitor 3-(5-fluoro-1H-indol-3-yl)pyrrolidine-2,5-dione (EOS200271/PF-06840003) and its characterization as a potential clinical candidate. J Med Chem (2017) 60(23):9617–29. doi: 10.1021/acs.jmedchem.7b00974 29111717

[127] Gomes B Driessens G Bartlett D Cai D Cauwenberghs S Crosignani S. Characterization of the selective indoleamine 2,3-dioxygenase-1 (IDO1) catalytic inhibitor EOS200271/PF-06840003 supports IDO1 as a critical resistance mechanism to PD-(L)1 blockade therapy. Mol Cancer Ther (2018) 17(12):2530–42. doi: 10.1158/1535-7163.MCT-17-1104 30232146

[128] Sonpavde G Necchi A Gupta S Steinberg GD Gschwend JE van der Heijden MS. ENERGIZE: a Phase III study of neoadjuvant chemotherapy alone or with nivolumab with/without linrodostat mesylate for muscle-invasive bladder cancer. Future Oncol (2020) 16(2):4359–68. doi: 10.2217/fon-2019-0611 31823654

[129] Yap TA Sahebjam S Hong DS Chiu VK Yilmaz E Efuni S. First-in-human study of KHK2455, a long-acting, potent and selective indoleamine 2,3-dioxygenase 1 (IDO-1) inhibitor, in combination with mogamulizumab (Moga), an anti-CCR4 monoclonal antibody, in patients (pts) with advanced solid tumors. J Clin Oncol (2018) 36(15_suppl):3040. doi: 10.1200/JCO.2018.36.15_suppl.3040 29847297

[130] Kotecki N Vuagnat P O'Neil BH Jalal S Rottey S Prenen H. A phase I study of an IDO-1 inhibitor (LY3381916) as monotherapy and in combination with an anti-PD-L1 antibody (LY3300054) in patients with advanced cancer. J Immunother (2021) 44(7):264–75. doi: 10.1097/CJI.0000000000000368 33928928

[131] Reardon DA Desjardins A Rixe O Cloughesy T Alekar S Williams JH. A phase 1 study of PF-06840003, an oral indoleamine 2,3-dioxygenase 1 (IDO1) inhibitor in patients with recurrent Malignant glioma. Invest New Drugs (2020) 38(6):1784–95. doi: 10.1007/s10637-020-00950-1 32436060

[132] Zhang L Cherney EC Zhu X Lin TA Gullo-Brown J Maley D. Discovery of imidazopyridines as potent inhibitors of indoleamine 2,3-dioxygenase 1 for cancer immunotherapy. ACS Med Chem Lett (2021) 12(3):494–501. doi: 10.1021/acsmedchemlett.1c00014 33738077 PMC7958151

[133] Steeneck C Kinzel O Anderhub S Hornberger M Pinto S Morschhaeuser B. Discovery and optimization of substituted oxalamides as novel heme-displacing IDO1 inhibitors. Bioorg Med Chem Lett (2021) 33:127744. doi: 10.1016/j.bmcl.2020.127744 33333163

[134] Hamilton MM Mseeh F McAfoos TJ Leonard PG Reyna NJ Harris AL. Discovery of IACS-9779 and IACS-70465 as potent inhibitors targeting indoleamine 2,3-dioxygenase 1 (IDO1) apoenzyme. J Med Chem (2021) 64(15):11302–29. doi: 10.1021/acs.jmedchem.1c00679 34292726

[135] Bollu LR Bommi PV Monsen PJ Zhai L Lauing KL Bell A. Identification and characterization of a novel indoleamine 2,3-dioxygenase 1 protein degrader for glioblastoma. J Med Chem (2022) 65(23):15642–62. doi: 10.1021/acs.jmedchem.2c00771 PMC9743093 36410047

[136] Shi D Wu X Jian Y Wang J Huang C Mo S. USP14 promotes tryptophan metabolism and immune suppression by stabilizing IDO1 in colorectal cancer. Nat Commun (2022) 13(1):5644. doi: 10.1038/s41467-022-33285-x 36163134 PMC9513055

[137] Winters M DuHadaway JB Pham KN Lewis-Ballester A Badir S Wai J. Diaryl hydroxylamines as pan or dual inhibitors of indoleamine 2,3-dioxygenase-1, indoleamine 2,3-dioxygenase-2 and tryptophan dioxygenase. Eur J Med Chem (2019) 162:455–64. doi: 10.1016/j.ejmech.2018.11.010 PMC6318801 30469041

[138] Rohrig UF Majjigapu SR Caldelari D Dilek N Reichenbach P Ascencao K. 1,2,3-Triazoles as inhibitors of indoleamine 2,3-dioxygenase 2 (IDO2). Bioorg Med Chem Lett (2016) 26(17):4330–3. doi: 10.1016/j.bmcl.2016.07.031 27469130

[139] Bakmiwewa SM Fatokun AA Tran A Payne RJ Hunt NH Ball HJ. Identification of selective inhibitors of indoleamine 2,3-dioxygenase 2. Bioorg Med Chem Lett (2012) 22(24):7641–6. doi: 10.1016/j.bmcl.2012.10.010 23122865

[140] He X He G Chu Z Wu H Wang J Ge Y. Discovery of the first potent IDO1/IDO2 dual inhibitors: A promising strategy for cancer immunotherapy. J Med Chem (2021) 64(24):17950–68. doi: 10.1021/acs.jmedchem.1c01305 34854662

[141] Kozlova A Frederick R. Current state on tryptophan 2,3-dioxygenase inhibitors: a patent review. Expert Opin Ther Pat (2019) 29(1):11–23. doi: 10.1080/13543776.2019.1556638 30526149

[142] Pham KN Lewis-Ballester A Yeh SR. Structural basis of inhibitor selectivity in human indoleamine 2,3-dioxygenase 1 and tryptophan dioxygenase. J Am Chem Soc (2019) 141(47):18771–9. doi: 10.1021/jacs.9b08871 PMC7366343 31682426

[143] Tijono SM Palmer BD Tomek P Flanagan JU Henare K Gamage S. Evaluation of novel inhibitors of tryptophan dioxygenases for enzyme and species selectivity using engineered tumour cell lines expressing either murine or human IDO1 or TDO2. Pharm (Basel) (2022) 15(9):1–19. doi: 10.3390/ph15091090 PMC9501369 36145311

[144] Oweira H Lahdou I Mehrle S Khajeh E Nikbakhsh R Ghamarnejad O. Kynurenine is the main metabolite of tryptophan degradation by tryptophan 2,3-dioxygenase in hepG2 tumor cells. J Clin Med (2022) 11(16):1–11. doi: 10.3390/jcm11164794 PMC9410271 36013032

[145] Cecchi M Mannini A Lapucci A Silvano A Lulli M Luceri C. Dexamethasone promotes a stem-like phenotype in human melanoma cells *via* tryptophan 2,3 dioxygenase. Front Pharmacol (2022) 13:911019. doi: 10.3389/fphar.2022.911019 35847038 PMC9280025

[146] Cecchi M Paccosi S Silvano A Eid AH Parenti A. Dexamethasone induces the expression and function of tryptophan-2-3-dioxygenase in SK-MEL-28 melanoma cells. Pharm (Basel) (2021) 14(3):1–16. doi: 10.3390/ph14030211 PMC7998133 33806305

[147] Paccosi S Cecchi M Silvano A Parenti A. Different effects of tryptophan 2,3-dioxygenase inhibition on SK-Mel-28 and HCT-8 cancer cell lines. J Cancer Res Clin Oncol (2020) 146(12):3155–63. doi: 10.1007/s00432-020-03351-2 PMC7679327 32776284

[148] Chuang TD Quintanilla D Boos D Khorram O. Tryptophan catabolism is dysregulated in leiomyomas. Fertil Steril (2021) 116(4):1160–71. doi: 10.1016/j.fertnstert.2021.05.081 PMC8787760 34116832

[149] Bostian AC Maddukuri L Reed MR Savenka T Hartman JH Davis L. Kynurenine signaling increases DNA polymerase kappa expression and promotes genomic instability in glioblastoma cells. Chem Res Toxicol (2016) 29(1):101–8. doi: 10.1021/acs.chemrestox.5b00452 PMC4718841 26651356

[150] Zhang R Wang Y Liu D Luo Q Du P Zhang H. Sodium tanshinone IIA sulfonate as a potent IDO1/TDO2 dual inhibitor enhances anti-PD1 therapy for colorectal cancer in mice. Front Pharmacol (2022) 13:870848. doi: 10.3389/fphar.2022.870848 35571116 PMC9091350

[151] Gyulveszi G Fischer C Mirolo M Stern M Green L Ceppi M. Abstract LB-085: RG70099: A novel, highly potent dual IDO1/TDO inhibitor to reverse metabolic suppression of immune cells in the tumor micro-environment. Cancer Res (2016) 76:LB–085. doi: 10.1158/1538-7445.AM2016-LB-085

[152] Gullapalli S Roychowdhury A Khaladkar T Sawargave S Janrao R Kalhapure V. Abstract 1701: EPL-1410, a novel fused heterocycle based orally active dual inhibitor of IDO1/TDO2, as a potential immune-oncology therapeutic. Cancer Res (2018) 78:1701. doi: 10.1158/1538-7445.AM2018-1701

[153] Naing A Eder JP Piha-Paul SA Gimmi C Hussey E Zhang S. Preclinical investigations and a first-in-human phase I trial of M4112, the first dual inhibitor of indoleamine 2,3-dioxygenase 1 and tryptophan 2,3-dioxygenase 2, in patients with advanced solid tumors. J Immunother Cancer (2020) 8(2):1–10. doi: 10.1136/jitc-2020-000870 PMC7449315 32843490

[154] Han Q Robinson H Cai T Tagle DA Li J. Structural insight into the inhibition of human kynurenine aminotransferase I/glutamine transaminase K. J Med Chem (2009) 52(9):2786–93. doi: 10.1021/jm9000874 PMC2844090 19338303

[155] Jacobs KR Castellano-Gonzalez G Guillemin GJ Lovejoy DB. Major developments in the design of inhibitors along the kynurenine pathway. Curr Med Chem (2017) 24(23):2471–95. doi: 10.2174/0929867324666170502123114 PMC5748880 28464785

[156] Lemos H Mohamed E Ou R McCardle C Zheng X McGuire K. Co-treatments to boost IDO activity and inhibit production of downstream catabolites induce durable suppression of experimental autoimmune encephalomyelitis. Front Immunol (2020) 11:1256. doi: 10.3389/fimmu.2020.01256 32625215 PMC7311583

[157] Pérez de la Cruz G Pérez de la Cruz V Navarro Cossio J Vázquez Cervantes GI Salazar A Orozco Morales M. Kynureninase promotes immunosuppression and predicts survival in glioma patients: in silico data analyses of the chinese glioma genome atlas (CGGA) and of the cancer genome atlas (TCGA). Pharm (Basel) (2023) 16(3):369. doi: 10.3390/ph16030369 PMC10051585 36986469

[158] Phillips RS. Structure, mechanism, and substrate specificity of kynureninase. Biochim Biophys Acta (2011) 1814(11):1481–8. doi: 10.1016/j.bbapap.2010.12.003 PMC3102132 21167323

[159] Kasper SH Bonocora RP Wade JT Musah RA Cady NC. Chemical inhibition of kynureninase reduces pseudomonas aeruginosa quorum sensing and virulence factor expression. ACS Chem Biol (2016) 11(4):1106–17. doi: 10.1021/acschembio.5b01082 26785289

[160] Drysdale MJ Reinhard JF. S-aryl cysteine S,S-dioxides as inhibitors of mammalian kynureninase. Bioorg Med Chem Lett (1998) 8(2):133–8. doi: 10.1016/S0960-894X(97)10209-8 9871640

[161] Bender DA Wynick D. Inhibition of kynureninase (L-kynurenine hydrolase, EC 3 . 7. 1 . 3) by oestrone sulphate: an alternative explanation for abnormal results of tryptophan load tests in women receiving oestrogenic steroids. Br J Nutr (1981) 45(2):269–75. doi: 10.1079/bjn19810103 7213582

[162] Chiarugi A Carpenedo R Molina MT Mattoli L Pellicciari R Moroni F. Comparison of the neurochemical and behavioral effects resulting from the inhibition of kynurenine hydroxylase and/or kynureninase. J Neurochem (1995) 65(3):1176–83. doi: 10.1046/j.1471-4159.1995.65031176.x 7643095

[163] Zhang Y Wang L Ren W. Blast-related traumatic brain injury is mediated by the kynurenine pathway. Neuroreport (2022) 33(13):569–76. doi: 10.1097/WNR.0000000000001817 35894672

[164] Yang Q Hao J Chi M Wang Y Xin B Huang J. Superior antitumor immunotherapy efficacy of kynureninase modified CAR-T cells through targeting kynurenine metabolism. Oncoimmunology (2022) 11(1):2055703. doi: 10.1080/2162402X.2022.2055703 35355679 PMC8959528

[165] Zeng Z Zhang C Li J Cui D Jiang Y Pu K. Activatable polymer nanoenzymes for photodynamic immunometabolic cancer therapy. Advanced Materials (2021) 33(4):2007247. doi: 10.1002/adma.202007247 33306220

[166] Hutchinson JP Rowland P Taylor MRD Christodoulou EM Haslam C Hobbs CI. Structural and mechanistic basis of differentiated inhibitors of the acute pancreatitis target kynurenine-3-monooxygenase. Nat Commun (2017) 8:15827. doi: 10.1038/ncomms15827 28604669 PMC5477544

[167] Beaumont V Mrzljak L Dijkman U Freije R Heins M Rassoulpour A. The novel KMO inhibitor CHDI-340246 leads to a restoration of electrophysiological alterations in mouse models of Huntington's disease. Exp Neurol (2016) 282:99–118. doi: 10.1016/j.expneurol.2016.05.005 27163548

[168] Smith JR Jamie JF Guillemin GJ. Kynurenine-3-monooxygenase: a review of structure, mechanism, and inhibitors. Drug Discovery Today (2016) 21(2):315–24. doi: 10.1016/j.drudis.2015.11.001 26589832

[169] Mole DJ Webster SP Uings I Zheng X Binnie M Wilson K. Kynurenine-3-monooxygenase inhibition prevents multiple organ failure in rodent models of acute pancreatitis. Nat Med (2016) 22(2):202–9. doi: 10.1038/nm.4020 PMC4871268 26752518

[170] Gao J Yao L Xia T Liao X Zhu D Xiang Y. Biochemistry and structural studies of kynurenine 3-monooxygenase reveal allosteric inhibition by Ro 61-8048. FASEB J (2018) 32(4):2036–45. doi: 10.1096/fj.201700397RR 29208702

[171] Fernando D Dimelow R Gorey C Zhu X Muya C Parker C. Assessment of the safety, pharmacokinetics and pharmacodynamics of GSK3335065, an inhibitor of kynurenine monooxygenase, in a randomised placebo-controlled first-in-human study in healthy volunteers. Br J Clin Pharmacol (2022) 88(2):865–70. doi: 10.1111/bcp.15010 34327739

[172] Berg M Polyzos KA Agardh H Baumgartner R Forteza MJ Kareinen I. 3-Hydroxyanthralinic acid metabolism controls the hepatic SREBP/lipoprotein axis, inhibits inflammasome activation in macrophages, and decreases atherosclerosis in Ldlr-/- mice. Cardiovasc Res (2020) 116(12):1948–57. doi: 10.1093/cvr/cvz258 PMC7519886 31589306

[173] Agrawal VK Sohgaura R Khadikar PV. QSAR study on inhibition of brain 3-hydroxy-anthranilic acid dioxygenase (3-HAO): a molecular connectivity approach. Bioorg Med Chem (2001) 9(12):3295–9. doi: 10.1016/S0968-0896(01)00242-5 11711305

[174] Linderberg M Hellberg S Björk S Gotthammar B Högberg T Persson K. Synthesis and QSAR of substituted 3-hydroxyanthranilic acid derivatives as inhibitors of 3-hydroxyanthranilic acid dioxygenase (3-HAO). Eur J Medicinal Chem (1999) 34(9):729–44. doi: 10.1016/S0223-5234(99)00220-2

[175] Braidy N Guillemin GJ Grant R. Effects of kynurenine pathway inhibition on NAD metabolism and cell viability in human primary astrocytes and neurons. Int J Tryptophan Res (2011) 4:29–37. doi: 10.4137/IJTR.S7052 22084601 PMC3195218

[176] Modoux M Rolhion N Mani S Sokol H. Tryptophan metabolism as a pharmacological target. Trends Pharmacol Sci (2021) 42(1):60–73. doi: 10.1016/j.tips.2020.11.006 33256987

[177] Ahern TP Spector LG Damkier P Öztürk Esen B Ulrichsen SP Eriksen K. Medication-associated phthalate exposure and childhood cancer incidence. J Natl Cancer Inst (2022) 114(6):885–94. doi: 10.1093/jnci/djac045 PMC9194627 35179607

[178] Guo T Meng X Liu X Wang J Yan S Zhang X. Associations of phthalates with prostate cancer among the US population. Reprod Toxicol (2023) 116:108337. doi: 10.1016/j.reprotox.2023.108337 36646329

[179] Kluwe WM. Carcinogenic potential of phthalic acid esters and related compounds: structure-activity relationships. Environ Health Perspect (1986) 65:271–8. doi: 10.1289/ehp.8665271 PMC1474699 3709453

[180] Dunn GP Bruce AT Ikeda H Old LJ Schreiber RD. Cancer immunoediting: from immunosurveillance to tumor escape. Nat Immunol (2002) 3(11):991–8. doi: 10.1038/ni1102-991 12407406

[181] Liang F Wang GZ Wang Y Yang YN Wen ZS Chen DN. Tobacco carcinogen induces tryptophan metabolism and immune suppression *via* induction of indoleamine 2,3-dioxygenase 1. Signal Transduct Target Ther (2022) 7(1):311. doi: 10.1038/s41392-022-01127-3 36068203 PMC9448807

[182] Sweeney C Lazennec G Vogel CFA. Environmental exposure and the role of AhR in the tumor microenvironment of breast cancer. Front Pharmacol (2022) 13:1095289. doi: 10.3389/fphar.2022.1095289 36588678 PMC9797527

[183] Shen X Zhao Y Liu G Zhou HL Fan J Zhang L. Upregulation of programmed death ligand 1 by liver kinase B1 and its implication in programmed death 1 blockade therapy in non-small cell lung cancer. Life Sci (2020) 256:117923. doi: 10.1016/j.lfs.2020.117923 32522567

[184] Zhu B Tang L Chen S Yin C Peng S Li X. Targeting the upstream transcriptional activator of PD-L1 as an alternative strategy in melanoma therapy. Oncogene (2018) 37(36):4941–54. doi: 10.1038/s41388-018-0314-0 29786078

[185] Feng J Read OJ Dinkova-Kostova AT. Nrf2 in TIME: the emerging role of nuclear factor erythroid 2-related factor 2 in the tumor immune microenvironment. Mol Cells (2023) 46(3):142–52. doi: 10.14348/molcells.2023.2183 PMC10070167 36927604

